# RNA-Based Therapeutic Strategies in Multiple Myeloma: From Molecular Targets to Delivery and Clinical Translation

**DOI:** 10.3390/ijms27020843

**Published:** 2026-01-14

**Authors:** Maksim V. Baranov, Igor Shalik, Angela Tsvetkova, Anna Streltsova, Dmitriy Ovcharenko, Roman Ivanov, Vasiliy Reshetnikov

**Affiliations:** 1 Translational Medicine Research Center, Sirius University of Science and Technology, 354340 Sochi, Russia; 2 Independent Researcher, Austin, TX 78748, USA

**Keywords:** multiple myeloma, RNA-therapeutics, lipid nanoparticles, cell therapies

## Abstract

Multiple myeloma (MM) is a challenging hematologic malignancy characterized by clonal plasma cell proliferation, often leading to significant morbidity and mortality worldwide. Despite advances in chemotherapy and CAR-T therapies, MM remains incurable due to tumor heterogeneity, immune evasion, and microenvironment remodeling—exacerbated by toxicities like cytokine release syndrome and myelosuppression. This urgent unmet need demands innovative strategies. In this review, we assess cutting-edge RNA-based therapeutics for MM modulation, drawing on preclinical and clinical evidence on modalities including mRNA vaccines, small interfering RNAs (siRNAs), antisense oligonucleotides (ASOs), and microRNA (miRNA) mimics/inhibitors. We further explore RNA-engineered cell therapies, such as transient CAR-T platforms and lipid nanoparticle-delivered systems targeting the bone marrow niche. By integrating these insights, we underscore RNA technologies’ transformative potential to achieve durable remissions, overcome resistance, and reduce costs—paving the way for personalized, safer treatments in refractory MM.

## 1. Introduction

**Figure 1:**
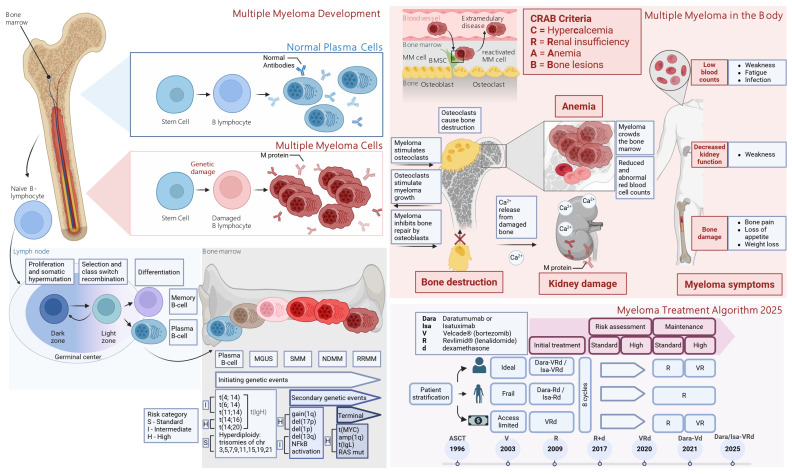
Overview of multiple myeloma (MM) pathogenesis, systemic manifestations, and current treatment landscape. Schematic representation of MM initiation, progression, and therapeutic management. (**Left**) Normal B-cell differentiation and plasma-cell maturation within the germinal center and bone marrow are compared with malignant transformation leading to monoclonal plasma-cell expansion. Genetic and epigenetic lesions drive progression through precursor stages—monoclonal gammopathy of undetermined significance (MGUS) and smoldering MM (SMM)—toward newly diagnosed (NDMM) and relapsed/refractory MM (RRMM). Common initiating cytogenetic abnormalities (e.g., t(4;14), t(6;14), t(11;14), t(14;16), t(14;20)) and secondary mutations (MYC, RAS, NF-κB activation) define molecular risk subgroups. (**Top right**) The clinical (CRAB) features—C, hypercalcemia; R, renal insufficiency; A, anemia; B, bone lesions—summarize major organ involvement. Myeloma-derived cytokines promote osteoclast activation, osteoblast inhibition, and calcium release, resulting in bone destruction, anemia from marrow infiltration, and renal damage from M-protein accumulation. (**Bottom right**) The updated 2025 MM treatment algorithm integrates patient stratification (transplant-eligible, frail, or access-limited) with quadruplet induction regimens (e.g., Dara-VRd or Isa-VRd), followed by autologous stem-cell transplantation (ASCT) and maintenance therapy. Current standards emphasize CD38-targeting antibodies, immunomodulatory drugs (IMiDs), and proteasome inhibitors, with RNA- and cell-based strategies emerging for refractory disease. Created in BioRender. Baranov, M. (2025) https://BioRender.com/sq7ets4 (access on 5 December 2025).

Multiple myeloma (MM) accounts for over 10% [1,2] of hematologic malignancies globally, claiming more than 100,000 lives annually [1,3]. Multiple myeloma (MM) is a clonal plasma-cell malignancy that predominantly arises within the bone marrow (BM) [4,5,6,7], with occasional extramedullary involvement (Figure 1) [4,6,8]. Disease biology reflects extensive inter- and intra-patient heterogeneity driven by cooperating genetic lesions and microenvironmental cues [6]. The BM niche fosters malignant growth and immune evasion through stromal and immune cell interactions and soluble mediators, which together promote bone destruction, angiogenesis, immunosuppression, and therapy resistance [6,7].

Dysregulation at the RNA level, together with epigenetic remodeling, defines MM phenotypes. For instance, upregulated long non-coding RNAs (lncRNAs), such as MALAT1 and circular RNAs (circRNA), like circ_0007841 (acting via miR-129-5p/JAG1), alter transcriptional networks, enhance immune escape mediated by the tumor microenvironment (TME), and foster therapy resistance [9,10,11,12,13,14,15,16]. Emerging evidence also reveals sex-specific dysregulation of exosomal non-coding RNAs (ncRNAs) in MM progression. This further links these alterations to metabolic reprogramming and evasion of therapeutic interventions [17]. Single-cell studies across monoclonal gammopathy of undetermined significance (MGUS)/smoldering multiple myeloma (SMM) delineate early transcriptomic and ncRNA alterations that predict malignant evolution [11,12,13]. Exosomal miRNAs and metabolic shifts contribute to subclonal diversification [18,19,20]. These findings, supported by large-scale scRNA-seq datasets (e.g., over 6 million cells from MM and precursors), reveal evolving immune dysregulation from MGUS to active MM. This includes myeloid suppressor cell expansion and T-cell exhaustion [12,21].

Globally, MM accounted for approximately 188,000 new cases and 121,000 deaths in 2022. This represents ~1% of all cancers [3] and 10–15% of hematologic malignancies worldwide [3,6,7]. Incidence and mortality have risen substantially since 1990, with age-standardized rates increasing from ~1.2 to 1.7 per 100,000 (1990–2021). Aging populations and high body-mass index are key drivers [22,23,24]. Projections estimate a 71% rise in incidence and 79% in mortality by 2045 without interventions [3]. In the U.S., incidence has been ~7.3 per 100,000 (2013–2022) with ongoing mortality improvements [25,26,27]. Marked disparities persist: non-Hispanic Black populations experience 2–3× higher incidence and mortality than non-Hispanic Whites [25,26,28,29,30,31]. The median diagnosis age is ~69 years, and the COVID-19 era further strained outcomes through diagnosis delays and excess morbidity/mortality [26,32,33,34,35]. Even in 2025, post-Omicron data show elevated hospitalization risks (e.g., higher intensive care unit admissions) and suboptimal vaccine responses in MM patients, underscoring ongoing vulnerabilities [36,37,38,39].

### 1.1. Current Standard of Care and Unmet Needs

**Figure 2:**
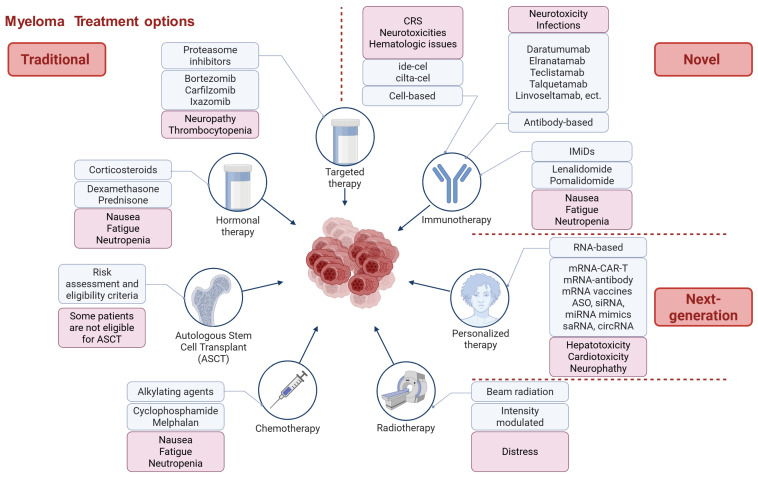
Evolution of therapeutic strategies in multiple myeloma (MM): from traditional regimens to next-generation RNA-based modalities. This schematic illustrates the main therapeutic categories currently employed or emerging in MM management. Traditional approaches include chemotherapy (e.g., cyclophosphamide, melphalan), hormonal therapy (dexamethasone, prednisone), autologous stem cell transplantation (ASCT) for eligible patients, and radiotherapy for palliative symptom control. Targeted therapies, such as proteasome inhibitors (bortezomib, carfilzomib, ixazomib) and immunomodulatory drugs (IMiDs) (lenalidomide, pomalidomide), form the backbone of standard-of-care regimens, often combined with monoclonal antibodies (daratumumab, elranatamab, teclistamab, talquetamab, linvoseltamab). Created in BioRender. Baranov, M. (2025) https://BioRender.com/ng5qi8c (access on 5 December 2025).

The standard of care (SOC) for multiple myeloma (MM) is based on combinations of immunomodulatory drugs (IMiDs), proteasome inhibitors (PIs), and monoclonal antibodies (mAbs), with autologous stem cell transplantation (ASCT)—high-dose chemotherapy followed by reinfusion of the patient’s own stem cells—offered as consolidation to eligible patients with newly diagnosed MM (NDMM). Patients with relapsed or refractory MM (RRMM) require subsequent, line-dependent regimens. Over the past decade, MM therapy has shifted from conventional chemotherapy toward risk-adapted strategies incorporating IMiDs, PIs, anti-CD38 mAbs, and cellular therapies (Figure 2), as reflected in recent National Comprehensive Cancer Network (NCCN) and American Society of Clinical Oncology (ASCO) 2025 guidelines [40,41]. For transplant-eligible NDMM, quadruplet induction with daratumumab (Dara)-bortezomib (V)-lenalidomide (R)-dexamethasone (d) (Dara-VRd) is now preferred. This is followed by ASCT and lenalidomide maintenance. The PERSEUS (NCT03710603) showed superior depth and durability of response [40,41,42]. Updated 2025 analyses confirm sustained MRD negativity and improved PFS with Dara-VRd, reinforcing it as SOC [43]. In transplant-ineligible NDMM, Dara-Rd or Dara-VMP remain standards, with Isa-VRd added per recent NCCN updates; continuous Rd is appropriate for frail patients [40]. These regimens extend progression-free survival. However, limitations like persistent MRD from antigen escape and TME-mediated resistance remain. This highlights the need for novel therapeutic approaches.

In relapsed/refractory MM (RRMM), treatment is line-dependent, with bispecific antibodies (BsAbs) (e.g., teclistamab [BCMA × CD3], talquetamab [GPRC5D × CD3], elranatamab [BCMA × CD3]) and CAR-T (idecabtagene vicleucel [ide-cel], ciltacabtagene autoleucel [cilta-cel]) therapies have reshaped options for triple-class refractory disease [40,44]. Recent approvals include linvoseltamab (Regeneron), a BCMA × CD3 BsAb. It received accelerated FDA approval 2 July 2025, ORR 70.9% in RRMM (49.6% CR) in the LINKER-MM1 phase 1/2 trial [45]. Other BsAbs in development (e.g., ABBV-383, cevostamab, forimtamig) are in phase 1–3 trials but not yet approved [46,47,48,49]. ABBV-383 entered Phase 3 (Cervino) in 2024 with ongoing enrollment. Cevostamab shows promise as post-CAR-T consolidation in Phase 1/2. Forimtamig remains in Phase 1 [50]. Trispecific antibodies (e.g., JNJ-79635322, targeting BCMA, GPRC5D, and CD3) are in early clinical trials and show promise for reducing antigen escape, but no approvals yet [51]. GPRC5D-targeted CAR-T therapies (e.g., BMS-986393, OriCAR-017) are in phase 1 trials, with no regulatory approvals as of November 2025 [52,53,54]. BMS-986393 and OriCAR-017 continue in Phase 1/2 with encouraging early safety/efficacy data but no approvals. For high-risk MM (e.g., del(17p), t(4;14)), intensified quadruplets are used, while smoldering MM (SMM) management focuses on monitoring, with Dara monotherapy or Rd for high-risk to delay progression [40]. ASH 2024 and EHA 2025 highlighted Circio’s TG01 mutant RAS vaccine showing early clinical benefit, including stable disease or better in ~50% of evaluatable patients with high-risk SMM or MM harboring KRAS/NRAS mutations (interim Phase 1/2 data, NCT05841550) [55,56].

Despite these advances, unmet needs persist, particularly in overcoming resistance, immune evasion, and achieving durable remissions in RRMM and high-risk subtypes [40,51,57,58]. Nearly all patients relapse, with median PFS < 6 months in triple-class refractory RRMM [59], driven by antigen loss (e.g., BCMA/GPRC5D) and TME-mediated evasion [1,6,25,40,60,61,62,63,64]. High costs (>USD 400,000 for CAR-T/BsAbs) limit access, and toxicities, like cytokine release syndrome (CRS) and neurotoxicity, remain challenging [28,65]. Updates in 2025 emphasize cardiac toxicities from novel agents and strategies to mitigate financial burden through value-based care [66,67]. In SMM, preventive strategies are limited, with ~50% high-risk progressing within 2 years [68,69,70]. ASH 2024 and EHA 2025 highlighted gaps in early-relapse management and TME modulation.

Recent immunotherapeutic approaches, including CAR-T cells (ide-cel, cilta-cel) and bispecific antibodies, offer durable responses in relapsed/refractory MM but remain limited by cytokine release syndrome (CRS), neurotoxicity, and hematologic toxicities. Next-generation RNA-based therapies—such as mRNA-CAR-T, mRNA-encoded antibodies and vaccines, antisense oligonucleotides (ASOs), small interfering RNA (siRNA), microRNA mimics, self-amplifying RNA (saRNA), and circular RNA (circRNA)—represent a rapidly advancing class aiming to transiently reprogram immune or tumor cells without genomic integration. These strategies may overcome resistance and enable precision targeting of oncogenic transcripts or immunosuppressive pathways within the bone marrow microenvironment, but challenges remain regarding hepatotoxicity, cardiotoxicity, and scalability.

### 1.2. Signaling and the Tumor Microenvironment (TME) in MM

The clinical course of myeloma typically follows a stepwise trajectory, beginning with monoclonal gammopathy of undetermined significance (MGUS), transitioning through smoldering myeloma (SMM), and eventually progressing to symptomatic multiple myeloma (MM), with some patients developing extramedullary spread or plasma cell leukemia [6,71]. MM is characterized by the clonal expansion of malignant plasma cells in the bone marrow, driven by diverse genetic [71,72] and epigenetic [13] alterations that disrupt normal immune and hematopoietic functions (Figure 1).

Genetic alterations driving multiple myeloma (MM) include primary events like hyperdiploidy and recurrent translocations (e.g., t(11;14), t(4;14), t(14;16)), which initiate disease development, and secondary events, such as 1q gain, 13q and 17p deletions, and MYC translocations (t(18;14)), that contribute to progression and poor prognosis [71]. These changes disrupt key oncogenes and tumor suppressors (e.g., CCND1, FGFR3, TP53, MYC), leading to dysregulated proliferation, survival, and genomic instability [71]. Transcriptomic analysis of malignant plasma cells was performed from MM patients using CoMMpass and PADIMAC datasets. It revealed highly expressed genes, including both well-known MM-associated markers (e.g., MCL1, CXCR4, TNFRSF17, SDC1, SLAMF7, PTP4A3, XBP1). Lesser-known candidates (IFI6, IFITM1, SIK1, ALDOA, ATP5MF, ATP5ME, PSMB4) are absent or low in healthy plasma cells [72].

Epigenetic dysregulation in MM includes widespread enhancer remodeling, histone modification imbalances, and DNA methylation heterogeneity. These changes rewire transcriptional programs critical for tumor progression and therapy resistance. A key example is the t(4;14) translocation leading to NSD2 overexpression and aberrant H3K36me2 deposition, which reduces H3K27me3 and enhances chromatin accessibility across the genome [13]. Mutations in additional epigenetic regulators (e.g., TET2, DNMT3A, KDM6A) and dynamic enhancer rewiring further contribute to transcriptional plasticity and drug resistance, especially in relapsed/refractory disease [13]. Recent 2025 multi-omics studies highlight epigenetic drivers of antigen escape in immunotherapy-resistant MM, such as promoter methylation changes in BCMA and GPRC5D, underscoring the need for epigenome-targeted therapies [13,73].

Beyond genetic and epigenetic alterations, MM progression and therapy resistance are strongly driven by the bone marrow tumor microenvironment (TME) [6,64,71,74,75,76,77], which acts as both a tumor supportive [71] and immunosuppressive niche [6]. The TME includes stromal cells, endothelial cells, osteoblasts, osteoclasts, immune cells (e.g., T-regs, MDSCs, B-regs) [6,76,77,78,79]. It also involves exosomes and miRNA cargo. These elements reshape the local milieu to favor tumor growth, angiogenesis, immune escape, and metabolic reprogramming. MM-associated long non-coding RNAs (lncRNAs) also participate in remodeling the TME [14]. Notably, the immunosuppressive shift begins as early as MGUS [77], with a decline in cytotoxic memory T cells and accumulation of MDSCs, Tregs [6], or exhausted T-cells [80], and persists even through remission [71]. Single-cell multi-omics analyses from 2024–2025 confirm progressive TME alterations, including loss of granzyme K^+^ memory cytotoxic T cells during MGUS to SMM transition, leading to reduced immunosurveillance [81,82]. Bidirectional signaling between MM cells and the TME drives genomic instability and the emergence of drug-resistant subclones [71,83], highlighting the co-evolution of tumor and niche. These findings emphasize the necessity of therapeutic approaches that disrupt not only cell-intrinsic oncogenic programs but also TME-mediated protection and immune evasion [64,75,83].

MM cells exploit their bone marrow niche by interacting with mesenchymal stromal cells (MSCs), which promote the activation of several pro-survival signaling cascades—including IL-6/JAK/STAT, PI3K/AKT, NF-κB, and MAPK/ERK—contributing to drug resistance, immunoevasion, and proliferation [83]. Recent studies underscore the therapeutic importance of these pathways: PI3K/AKT/mTOR signaling, activated by IL-6 and IGF-1, supports MM cell growth and resistance, and its inhibition—using agents like mTOR inhibitors, temsirolimus [84], everolimus [85]; or AKT inhibitors, afuresertib [86] or resibufogenin [87]—show promise in relapsed/refractory settings [83]. Molecular studies have identified regulators like miR-25-3p and HMGB2, which modulate this pathway, offering new therapeutic potential [83]. RAS/MAPK signaling is frequently dysregulated due to KRAS/NRAS/BRAF mutations [88], especially in advanced disease stages [83,89], and targeted therapies are now under investigation in clinical trials [90]. RAS/RAF mutations occur in up to 61% of MM cases, often subclonally, with ongoing Phase 1/2 trials (e.g., mirdametinib + sirolimus for RAS-mutated RRMM, NCT06876142) showing early efficacy [91]. Persistent IL-6-mediated activation of the JAK/STAT3 axis correlates with poor prognosis [83], with inhibitors like siltuximab and ruxolitinib offering early clinical benefit [83,92]. Although siltuximab showed a trend toward delayed progression in high-risk SMM, the study did not meet its primary endpoint, suggesting a limited or modest effect of IL-6 blockade in this setting [93]. However, 2025 meta-analyses of SMM interventions confirm a ~60% reduction in progression risk with early treatments, including IL-6/JAK inhibitors [94,95]. In parallel, Wnt/β-catenin signaling, triggered by stromal cells, drives MM progression and bone disease [14,83,96], while NF-κB activation—observed in over 80% of patients [83]—facilitates immune escape and drug resistance, with ongoing efforts to identify specific upstream drivers like PPFIBP1 [97]. Cross-talk between these pathways further complicates treatment, but dual-pathway targeting strategies are emerging as rational combination approaches [83,98]. Additionally, metabolic adaptations, such as cuproptosis [99,100,101] and lipid metabolism reprogramming [18], represent novel vulnerabilities within the MM microenvironment. Studies from 2025 link cuproptosis to lncRNA regulation (e.g., MAMDC2-AS1 inducing it in RRMM) and prognostic signatures, highlighting copper homeostasis as a target for TME modulation [102,103,104]. These TME-driven pathways underscore the recalcitrant nature of MM, where genetic/epigenetic instability and immunosuppressive remodeling evade conventional therapies. RNA platforms, through targeted silencing or transient reprogramming, offer a precise means to disrupt these barriers, as explored in subsequent sections.

## 2. Overcoming Resistance, Immune Evasion, and High-Risk MM

Despite substantial advances in therapy—including proteasome inhibitors (PIs), immunomodulatory drugs (IMiDs) [60], monoclonal antibodies, and autologous stem cell transplantation (ASCT)—multiple myeloma (MM) remains largely incurable [4,6,7,105]. The introduction of bortezomib (PI) [105] (FDA approval 2003 [83]), lenalidomide [106,107] (FDA approval 2006 [83]), and pomalidomide [108] (FDA approval 2013 [83]), (IMiDs), and monoclonal antibodies, such as anti-CD38 daratumumab [108] and anti- SLAMF7 elotuzumab [105], has significantly extended progression-free and overall survival, especially when used in combination regimens [109,110] and post-ASCT maintenance settings [111]. Recent 2025 analyses confirm 5-year overall survival rates exceeding 60% overall, with medians ranging from 72–99 months across cohorts, though disparities persist in older or high-risk groups [112]. Median overall survival has increased from approximately 3 years two decades ago to 8–10 years in transplant-eligible patients [105]. Nevertheless, nearly all patients eventually relapse and develop resistance [8], with progressively shorter remission durations after each line of therapy [60]. High-risk subtypes, including those with adverse cytogenetic features (e.g., del(17p), t(4;14)) [71], tend to relapse rapidly and respond poorly to standard treatments [1,113].

Importantly, genetic analyses from the CoMMpass study have identified a particularly aggressive subset of newly diagnosed MM patients (6.1%) termed double-hit myeloma, defined by bi-allelic inactivation of the TP53 tumor suppressor gene in combination with 1q amplification—alterations detectable only via next-generation sequencing [114]. Updates from 2025 from CoMMpass and IMS refine double-hit prognostic models, integrating multi-omics for better risk stratification in high-risk multiple myeloma (HRMM) [115,116]. The disease’s genetic complexity, which evolves over decades from precursors, like MGUS and smoldering MM, further complicates curative strategies [1,6,11]. Resistance to key drug classes, including PIs, IMiDs, and CD38-targeting antibodies [117], remains a major barrier, particularly in triple-class refractory patients [60,65], where median progression-free survival may be as short as 5 months [1]. Although novel approaches, such as CAR-T cell therapies (ide-cel, cilta-cel) [60,118] and bispecific antibodies (e.g., teclistamab, talquetamab), offer promising responses in heavily pretreated populations [60,65,105], they have not eliminated the risk of relapse and are limited by cost and accessibility [28,60,65]. Finally, aggressive variants, such as extramedullary myeloma [119], which exhibit higher mutational burdens and poor response to conventional therapy [120], further underscore the persistent challenge of achieving durable remission in MM.

### 2.1. Antigen Escape and Antigen Loss in MM

**Figure 3:**
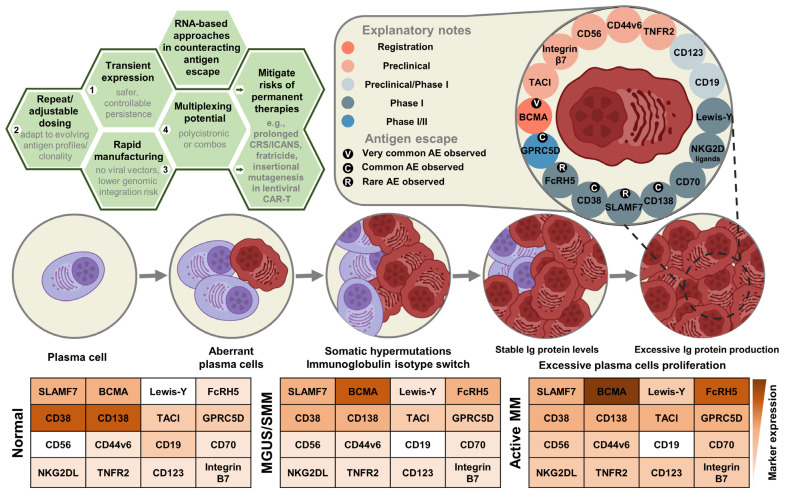
Antigen escape and antigen loss in multiple myeloma (MM): mechanisms and RNA-based countermeasures. Depicted immunophenotypic changes in plasma cells during MM progression (from normal to aberrant plasma cells, somatic hypermutations, stable/excessive Ig production), with surface marker expression varying by phase (e.g., loss of CD19 variable CD56/CD38, trend toward increasing BCMA/SLAMF7/FcRH5/GPRC5D). These markers serve as targets for antibody- or cell-based therapies but are prone to escape/loss via mechanisms like γ-secretase shedding (BCMA), heparanase/MMP shedding (CD138), trogocytosis/fratricide, or epigenetic silencing/mutations (promoter methylation, m6A modification). Antigen escape drives 30–70% of relapses in T-cell redirecting therapies (highest for BCMA > GPRC5D > CD38), with antigen escape events (AE) classified as very common (V), common (C), or rare (R) across preclinical/clinical phases. RNA-based approaches counter this through the following: (1) transient expression (safer, controllable persistence via mRNA/saRNA); (2) repeatable/adjustable dosing (adapt to clonality/evolving profiles); (3) faster manufacturing; (4) multiplexing (polycistronic mRNA CAR-T/bsAbs/TCEs for multi-targeting, e.g., BCMA + GPRC5D + CD38/FcRH5/SLAMF7). Abbreviations: AE, antigen escape; BCMA, B-cell maturation antigen; bsAbs, bispecific antibodies; CAR-T, chimeric antigen receptor T-cell; CD, cluster of differentiation; FcRH5, Fc receptor homolog 5; GPRC5D, G protein-coupled receptor class C group 5 member D; Ig, immunoglobulin; m6A, N6-methyladenosine; MM, multiple myeloma; MMP, matrix metalloproteinase; mRNA, messenger RNA; saRNA, self-amplifying RNA; SLAMF7, signaling lymphocytic activation molecule family member 7; TACI, transmembrane activator and CAML interactor; TCEs, T-cell engagers; TNFR2, tumor necrosis factor receptor 2. Note that NKG2D in the diagram refers to NKG2D/NKG2DL axis in MM; MM expresses NKG2D ligands (e.g., MICA, MICB, ULBP family members) on plasma cells, which are upregulated in malignant MM cells.

Antigen escape has emerged as a key resistance mechanism in immunotherapy for MM. In BCMA-targeted therapies, resistance mechanisms include biallelic loss of the TNFRSF17 gene encoding BCMA, clonal selection for BCMA-low or -negative subclones, and enzymatic shedding of BCMA by γ-secretase, which generates soluble BCMA (sBCMA) that impairs CAR-T cell binding [76,78] (Figure 3). Even moderately reduced antigen density can impair CAR-T cell activation due to signaling thresholds [62] (Figure 3). While antigen loss has been observed in clinical relapses after BCMA-targeted therapies, including [59] a meta-analysis in cohorts of over 2000 patients, it remains a relatively rare event (~4% of patients) and requires further validation in prospective large-scale studies [121,122,123,124]. Studies from 2025 confirm epigenetic repression (e.g., promoter methylation) as a reversible driver of BCMA/GPRC5D loss, amenable to hypomethylating agents like azacitidine in preclinical models [125,126,127].

In some patients, BCMA-negative relapse has been associated with upregulation of annexin A1 (ANXA1), a molecule that promotes myeloma growth and disrupts CAR-T targeting; preclinical inhibition of ANXA1 restores CAR-T sensitivity [128]. Similarly, resistance to GPRC5D-targeted agents can arise through gene deletion or epigenetic silencing of the GPRC5D locus. Notably, this epigenetic repression may be reversible using hypomethylating agents such as azacitidine (Figure 3) [63]. Epigenetic silencing of BCMA/GPRC5D has been documented in small clinical cohorts (e.g., post-CAR-T relapses), but these findings are primarily supported by preclinical models and have not been extensively validated in large patient populations (Figure 3) [63,64,121,127,129,130,131]. These findings support the development of next-generation multi-targeted immunotherapies—such as bispecific and trispecific antibodies that engage both BCMA and GPRC5D (e.g., JNJ-79635322 (JNJ-5322))—to mitigate antigen escape (Table 1) (Figure 3) [51]. Ongoing Phase 1/2 trials (e.g., dual BCMA/GPRC5D CAR-T) show reduced relapse from antigen escape, with sequential BCMA CAR-T feasible in refractory cases (Figure 3) [9,71,132,133].

**Figure 4:**
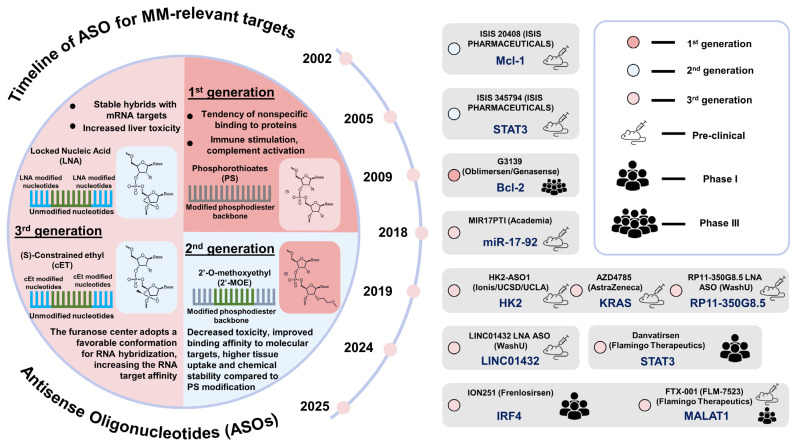
Timeline of antisense oligonucleotide (ASO) development for multiple myeloma (MM) and potentially applicable targets. This timeline illustrates the progression of ASO therapies from preclinical studies to ongoing clinical trials (2002–2025), including MM-specific and relevant targets (e.g., Mcl-1, STAT3, Bcl-2, miR-17-92, HK2, KRAS, RP11-350G8.5, LINC01432, IRF4, MALAT1). ASOs bind complementary mRNA sequences to induce RNase H-mediated degradation or sterically block translation, offering precise modulation of oncogenic drivers. Targets are MM-relevant: BCL2/IRF4/MALAT1/STAT3 overexpression correlates with poor prognosis/resistance; KRAS mutations drive progression; RP11-350G8.5/LINC01432 are upregulated in relapsed/refractory disease. Preclinical models show ASOs reduce myeloma cell viability, restore drug sensitivity (e.g., MALAT1 ASO reverses lenalidomide resistance via CD38 modulation), and inhibit glycolysis/apoptosis evasion, suggesting synergy with IMiDs/proteasome inhibitors. While few ASOs have reached MM-specific trials, their potential extends to untested but pathogenesis-relevant targets. Generations progressed as follows: 1st-gen (phosphorothioates/PS) offered stability but caused nonspecific binding, immune stimulation, and liver toxicity; 2nd-gen (2′-O-methyl/MOE) improved affinity/reduced toxicity via modified backbones; 3rd-gen (LNA/cEt) boosted potency, tissue uptake, and RNase H efficiency with chimeric designs (e.g., gapmers). Key milestones and outcomes: preclinical for HK2-ASO1 (HK2), AZD4785 (KRAS), RP11-350G8.5/LINC01432 LNA gapmers (lncRNAs); Phase III for Oblimersen (Bcl-2, encouraging responses but failed approval due to modest efficacy); Phase I for ION251 (IRF4, safety/tolerability assessed) and FTX-001 (MALAT1, favorable safety/target engagement, ongoing for solid tumors/AML-MDS with MM-preclinical signals). Mechanisms and validations (e.g., in vitro apoptosis, in vivo tumor reduction) emphasize the shift toward lncRNA/metabolic targets. Abbreviations: AML-MDS, acute myeloid leukemia-myelodysplastic syndrome; ASO, antisense oligonucleotide; Bcl-2/BCL2, B-cell lymphoma 2; cEt, constrained ethyl; HK2, hexokinase 2; IMiDs, immunomodulatory drugs; IRF4, interferon regulatory factor 4; KRAS, Kirsten rat sarcoma viral oncogene homolog; LNA, locked nucleic acid; lncRNAs, long non-coding RNAs; MALAT1, metastasis-associated lung adenocarcinoma transcript 1; Mcl-1, myeloid cell leukemia 1; miR-17-92, microRNA-17-92 cluster; MM, multiple myeloma; MOE, 2′-O-methoxyethyl; mRNA, messenger RNA; PS, phosphorothioate; RNase H, ribonuclease H; STAT3, signal transducer and activator of transcription.

RNA-based therapeutics offer advantages over lentiviral/retroviral CAR-T cells or recombinant protein bispecifics. These are limited to fixed specificity—often driving selective pressure for antigen-negative clones through persistent expression (in the case of viral CAR-T) or repeated dosing requirements (in the case of bispecifics). Transient mRNA-encoded CAR-T cells enable safe sequential administration (e.g., BCMA toGPRC5D/FcRH5/CD19 mRNA CAR upon resistance). They also support polycistronic designs encoding 2–3 antigens in one construct. This prevents escape via multi-target engagement and minimizes CRS/ICANS (mostly Grade 1–2). Outpatient dosing would be possible without lymphodepletion. Ex vivo electroporated autologous mRNA CAR-T cells, such as Cartesian Therapeutics’ Descartes-08 and Descartes-15 (anti-BCMA), have demonstrated an outstanding safety profile (repeated outpatient dosing, no lymphodepletion, virtually no high-grade CRS/ICANS or neurotoxicity) even in advanced clinical testing, with the platform now reaching Phase III in myasthenia gravis and delivering deep, durable responses in SLE (100% LLDAS at Month 3 in recent Phase 2 data). Cartesian’s strategic pivot toward autoimmune diseases—where depleting non-malignant, less heterogeneous plasma cells is biologically simpler and less prone to clonal evolution—further underscores the challenge of antigen escape in MM and the critical need for multi-targeted, adaptable RNA strategies in oncology. In vivo LNP-mRNA encoding CARs or multiplexed T-cell engagers (e.g., Moderna’s mRNA-2808 encoding BCMA/FcRH5/GPRC5D TCEs, now in Phase 1/2) bypass ex vivo manufacturing entirely, allowing rapid redosing/switching of targets with ultra-low toxicity and no genomic risk—ideal for monitoring/adapting to clonal evolution and antigen density shifts during progression (MGUS → SMM → MM → relapse; Figure 3). mRNA vaccines (e.g., BCMA-mRNA lipid nanoparticle vaccines or personalized neoantigen platforms) could generate broad polyclonal CD8^+^/CD4^+^ responses against multiple tumor-associated antigens simultaneously, dramatically lowering escape probability compared with monovalent therapies. Finally, it could potentially be an interesting solution to explore RNA-based therapies, such as siRNA/ASO or Cas13-based tools targeting epigenetic repressors (e.g., EZH2, LSD1, or KIAA1429), which would aim to reverse promoter silencing/m6A-mediated downregulation of BCMA/GPRC5D, restoring antigen density as rational combination pretreatment for established immunotherapies (Figure 3). However, these reversal strategies using siRNA, ASO, or Cas13 remain conceptual and are supported only by preclinical models, with no validation yet in clinical trials or large patient cohorts (Figure 4) [63,121,137,138]. These RNA modalities could thus transform antigen escape from an inevitable limitation into a manageable, adaptive challenge [131,139,140,141,142,143,144,145,146,147,148,149] (Figure 3). Having established the core biological barriers that render MM recalcitrant to current therapies, we now examine how RNA platforms are uniquely positioned to overcome each.

### 2.2. T Cell Exhaustion and the Immunosuppressive Niche

The immunosuppressive bone marrow microenvironment contributes significantly to immune evasion and therapeutic resistance in MM. Chronic antigen stimulation, secretion of immunosuppressive cytokines (e.g., IL-10, TGF-β), and interactions with regulatory immune cells promote T cell exhaustion and impair cytotoxic function. These features contribute to reduced durability of CAR-T cell and bispecific antibody responses [6]. Recent 2025 single-cell analyses reveal that T cells in newly diagnosed MM lack classic exhaustion hallmarks (e.g., no PD-1/TIM-3 overexpression, preserved antigen experience), but progressive dysfunction emerges in relapse, driven by metabolic amino acid deprivation and myeloid suppressor cells [150,151]. This exhaustion is often reversible post-therapy, with CAR-T reshaping the endogenous T cell landscape toward effector states [152]. Strategies, like checkpoint blockade (e.g., anti-PD-1) or bispecifics as bridging therapy, show promise in restoring T cell persistence in RRMM [153,154]. Reversing immune dysfunction—through immune checkpoint blockade, metabolic reprogramming, or myeloid cell modulation—remains an active area of research.

### 2.3. lncRNAs in Resistance and Progression: MALAT1, HOTAIR, RP11-350G8.5, and Others

Long non-coding RNAs (lncRNAs) are increasingly recognized as critical regulators of multiple myeloma (MM) progression, chemoresistance, and immune escape, with growing therapeutic relevance. Among these, MALAT1, HOTAIR, and RP11-350G8.5 have emerged as oncogenic lncRNAs with strong links to treatment resistance, disease progression, and poor prognosis [9,20,155,156].

MALAT1 (metastasis-associated lung adenocarcinoma transcript 1) is upregulated in relapsed and high-risk MM and associated with worse clinical outcomes [157,158]. Functionally, it enhances MM cell proliferation, chemoresistance (including to proteasome inhibitors), migration, and hypoxia survival [159,160,161]. Mechanistically, RNA-based therapeutics MALAT1 activates the EZH2/STAT3 axis, modulates the HIF-1α-KDM3A pathway under hypoxia [159], interacts with FAM46C to drive migration [161], and sponges tumor-suppressive miRNAs such as miR-125b, miR-1271-5p, miR-509-5p, miR-188-5p, and miR-15a/16 [158,162,163,164]. These diverse effects support MALAT1 as a key contributor to both chemoresistance and immune evasion. Investigations from 2025 studies confirm MALAT1’s role in lenalidomide resistance through epigenetic modulation of CD38 and induction of oxidative stress, where ASO targeting can restore drug sensitivity in preclinical experimental models [165] (Figure 4).

Recent RNA-based interventions include antisense oligonucleotides (ASOs) such as LNA gapmeRs, SWCNT-based constructs, and LNP-delivered RNA [166,167,168]. The first-in-class MALAT1 inhibitor FTX-001 has demonstrated promising target engagement, preclinical efficacy, and is advancing toward clinical trials [169].

HOTAIR (HOX transcript antisense RNA) contributes to MM pathogenesis through epigenetic repression of tumor suppressors and activation of NF-κB and JAK2/STAT3 signaling [170,171]. It exacerbates bone disease (especially in bisphosphonate induced osteonecrosis of the jaw (BRONJ) patients, [172]), and sponges miRNAs such as miR-23b-3p, miR-27b-3p, and miR-125b-5p [172]. Targeting strategies include ASO/siRNA knockdown [173,174], exosome-mediated delivery, and small molecules, such as AC1Q3QWB, which disrupts HOTAIR–PRC2 interaction [175].

RP11-350G8.5 is another oncogenic lncRNA overexpressed in MM, driving proliferation, survival, and treatment resistance. Its expression correlates with poor prognosis [176,177], and preclinical studies have validated its inhibition via RNA interference (RNAi) as a strategy to reduce MM burden. CRISPR-Cas9 screens in 2024–2025 confirm RP11-350G8.5 as a therapeutic target, with LNA ASOs reducing viability in MM cells [176,178].

Beyond these well-characterized lncRNAs, exosomal lncRNAs, such as NEAT1 and SNHG16, have been implicated in immune evasion and drug resistance, while AKAP12 and EMP1 serve as angiogenesis-linked lncRNAs integrated into immune-informed prognostic models [20,179]. New lncRNA candidates were discovered to control resistance to bortezomib and dexamethasone in MM [180]. NSUN2-modified exosomal MALAT1 drives RANKL-mediated bone destruction, positioning exosomes as both biomarkers and delivery vehicles for targeting such modifications [181]. Together, these findings underscore the promise of lncRNAs as both biomarkers and RNA-based therapeutic targets in MM.

### 2.4. Splicing and Epitranscriptomic Drivers in MM

RNA splicing and post-transcriptional modifications are increasingly recognized as critical components of MM pathogenesis, therapeutic resistance, and progression. Aberrant expression of splicing regulators, such as SRSF1 [182], CLK2, and USP39, alters mRNA isoform landscapes and enhances tumorigenic features. For example, CLK2 phosphorylates SRSF1, stabilizing RAE1 and promoting cell cycle progression, while its inhibition leads to RAE1 degradation and apoptosis [183]. Similarly, USP39 supports epithelial–mesenchymal transition (EMT) and drug resistance through stabilization of ZEB1 [184]. USP39 was identified as a critical survival factor, with siRNA knockdown impairing MM cell survival and migration [184]. Studies from 2025 reveal a MYC-USP39-SRSF1 axis driving splice-switching in MM, with high SRSF1 predicting poor prognosis and siRNA targeting showing anti-MM activity [185,186].

Moreover, SRSF1 mediates the oncogenic splicing of RBBP6, favoring the RBBP6-1 isoform over the tumor-suppressive RBBP6-3, with knockdown restoring p53 expression and impairing MM cell growth [187]. A 2018 study in Blood used siRNA to downregulate SRSF1, showing anti-MM activity [182].

In parallel, epitranscriptomic regulators contribute to RNA-level vulnerabilities in MM. METTL3, an m6A writer, enhances survival and drug resistance via the miR-182-5p/CAMK2N1 axis [188]. KIAA1429 stabilizes FOXM1 through YTHDF1, driving glycolysis and MM progression [189]. One study used siRNA to target KIAA1429, showing preclinical promise [189] (Figure 4). Recent epitranscriptomic reviews highlight m6A’s role in MM metabolism and resistance, with METTL3 overexpression linked to YY1 stability and miR-27 maturation [190,191]. On the acetylation front, NAT10 catalyzes ac4C modifications on XPO1 mRNA, supporting proteasome inhibitor resistance; dual inhibition of NAT10 and XPO1 restores drug sensitivity [192]. Similarly, NSUN2-mediated m5C modification of exosomal lncRNA MALAT1 promotes RANKL expression and bone destruction, highlighting m5C as an emerging epitranscriptomic driver in MM [181]. Studies show NAT10 promotes MM cell proliferation, with shRNA knockdown reducing growth [193]. Importantly, XPO1 is an FDA-approved druggable target in MM, with Selinexor—an XPO1 inhibitor—approved in 2019 for clinical use [83]. These findings position RNA modifiers as druggable drivers of MM, especially in relapsed/refractory settings.

miRNAs further shape the MM transcriptome by regulating autophagy, apoptosis, and stemness. miR-1343-3p suppresses autophagy by directly targeting ATG7 [194], while miR-138 promotes cancer stem cell survival via repression of PAX5, offering rationale for miRNA-based reprogramming [195]. miR-125b, often repressed by oncogenic lncRNAs like MALAT1 and HOTAIR, emerges as a central hub controlling immune and apoptotic networks [172]. miR-125b is a target for RNA-based therapy (lenti-shRNA mimics) in MM research, with preclinical evidence [193]. Updates from 2025 link miR-125b downregulation to MM progression via IRF4, with overexpression inhibiting growth in models [196,197].

RNA-based modulation of these pathways—via ASOs, shRNA, siRNA, or lipid nanoparticle (LNP)-delivered RNAi—represents a promising therapeutic strategy in MM.

## 3. RNA Platforms for Therapeutic Modulation in Multiple Myeloma

Despite advances in MM therapy, significant unmet needs persist: clonal heterogeneity and antigen escape drive inevitable relapse, immune evasion is promoted by MHC-I downregulation and immunosuppressive microenvironment remodeling [198,199], and current best-in-class immunotherapies are limited by severe toxicities (e.g., grade ≥ 3 CRS/ICANS in viral CAR-T), manufacturing complexity, and prohibitive costs that frequently exceed USD 400,000 per patient [28,65,200,201].

RNA-based therapeutics directly address many of these barriers by delivering controllable, and highly programmable gene expression without the risk of genomic integration [5]. These platforms span a broad spectrum of modalities—oligonucleotide-based gene silencing (siRNA, ASO, miRNA mimics/antagomirs), circular and self-amplifying RNA (circRNA, saRNA), mRNA-encoded cell engineering products (transient CAR-T, in vivo CAR programming, armored/secretable payloads), and mRNA vaccines (neoantigen, multi-antigen, or idiotype-based)—each with distinct biological mechanisms, manufacturing requirements, safety profiles, and clinical implementation strategies.

Because these modalities operate through fundamentally different mechanisms—direct gene silencing, transient cell engineering, or active induction of antitumor immunity—and face distinct translational hurdles in the immunosuppressive, bone-marrow-resident MM niche, they are discussed in separate subsections below.

### 3.1. Oligonucleotide-Based Therapies

#### 3.1.1. siRNA and Antisense Oligonucleotides (ASOs)

siRNAs silence oncogenic transcripts in MM through RNA interference, demonstrating promise against targets such as BCL2 family [202], MYC (NCT02110563, DCR-MYC, Dicerna Pharmaceuticals, Inc. [203], terminated in a phase Ib/II trial), IRF4 [204], CXCL13 [77]—all involved in MM proliferation, survival, and bone marrow retention. ASOs, including gapmeRs and single-walled carbon nanotube (SWCNT)-linked constructs, target both mRNAs and lncRNAs, such as MALAT1, to overcome drug resistance [166] (Table 2) (see Figure 4 for timeline and generations).

Advanced delivery systems, especially lipid-polymer nanoparticles, have been designed for bone marrow targeting. These improve RNA stability and in vivo efficacy [203,205]. Preclinical trials also demonstrate promising silencing of Cyclophilin A and potential for CD38- and BCMA-targeting siRNA LNPs [5,205]. As of the end of 2025, no siRNA- or ASO-based therapy has entered clinical testing specifically for multiple myeloma. The sole exception is the MALAT1-targeted ASO FTX-001 (Flamingo Therapeutics), which is advancing through preclinical development toward Phase 1 trials in advanced solid tumors (potent target engagement and safety demonstrated in preclinical models, as reported at EACR 2024, Abstract EACR2024-0240) [165,169]. Although no MM trial has been announced, recent preclinical studies in lenalidomide-resistant MM models with MALAT1-targeted ASOs show strong disease-relevant activity via CD38 upregulation and reduction of oxidative stress [165]. Among the RNA-based approaches summarized in Table 2, MALAT1-targeted ASOs (e.g., FTX-001) are realistically the closest to clinical translation, as the lead candidate is nearing Phase 1 in other cancers. Additionally, Flamingo Therapeutics’ lead candidate, danvatirsen (a STAT3-targeted ASO), is in a Phase 1 investigator-initiated trial (monotherapy followed by venetoclax combo) for relapsed/refractory AML and MDS (NCT05986240), with trial-in-progress data presented at ASH 2024 (Abstract 4265.5) [206]. Given STAT3’s association with adverse prognosis and treatment resistance in multiple myeloma, danvatirsen also holds potential relevance for MM. siRNA/ASO against MYC (prior Phase Ib/II terminated), BCL2 family, IRF4, SRSF1, USP39, KIAA1429, NAT10, and METTL3 remain solely at the preclinical stage. Most are supported only by in vitro or standard xenograft data, lacking both clinical-stage candidates and advanced delivery systems; therefore, they are currently considered early-stage or conceptual for direct therapeutic application. In contrast, approaches utilizing bone marrow- or plasma cell-targeted delivery platforms—such as CKAP5 siRNA in αCD38-tLNPs or Cyclophilin A siRNA in lipid-polymer nanoparticles—demonstrate substantially higher translational potential. This is because they directly address the major delivery barrier in MM, moving beyond the conceptual stage toward viable therapeutic strategies.

A growing set of MM-relevant targets have emerged in the last 1–2 years, including RNA splicing regulators (SRSF1 [182,187], USP39 [184]), epitranscriptomic enzymes (NAT10 [192,193], METTL3 [188], KIAA1429 [189]), and lncRNAs such as MALAT1 [167,168,169]. These targets regulate MM cell survival, EMT, glycolysis, and immune evasion—functions now being explored using siRNA or ASO-mediated silencing.

These findings reinforce siRNA and ASO as promising modalities to modulate MM progression, but clinical translation is limited by delivery hurdles, especially in the bone marrow niche. Optimization of LNP composition, conjugation with plasma cell-targeting ligands, and dual-delivery systems (e.g., siRNA + immunotherapy) are being explored to overcome these barriers [203,205,206,207] (Figure 4). While siRNA/ASO show promise in preclinical silencing of MM drivers, their comparative efficacy depends on optimized delivery systems, as analyzed in the following sections on nanoparticle platforms and clinical integration.

#### 3.1.2. miRNAs as Therapeutic Regulators

MicroRNAs (miRNAs) are 19–25-nucleotide non-coding RNAs that regulate gene expression post-transcriptionally by binding to complementary sequences in the 3′ untranslated regions (3′ UTRs) of target mRNAs, leading to translational repression or mRNA degradation. In multiple myeloma (MM), dysregulation of specific miRNAs drives disease progression by modulating critical pathways, including autophagy, apoptosis, cancer stemness, drug resistance, angiogenesis, and immune regulation.

For instance, miR-1343-3p targets ATG7, a key autophagy-related gene, suppressing autophagic flux in MM cells and impairing their adaptation to endoplasmic reticulum (ER) stress and proteotoxicity [194]. Similarly, miR-138 promotes MM cancer stem cell survival by repressing PAX5, a tumor suppressor transcription factor; its inhibition via antagomirs restores PAX5 expression, reducing clonogenicity and drug resistance [195]. Tumor-suppressive miRNAs, such as miR-125b and miR-509-5p, are sequestered by long non-coding RNAs (lncRNAs), like MALAT1 and HOTAIR, which act as competing endogenous RNAs (ceRNAs), enhancing oncogenic signaling [158,162,172,193]. Other tumor-suppressive miRNAs, including miR-15a, f, and miR-34a, target BCL2 and AURKA to promote apoptosis and inhibit proliferation, while oncogenic miRNAs (oncomiRs) like miR-21 and miR-221/222 drive drug resistance by targeting PTEN and other pathways [208,209,210,211,212].

These insights highlight the therapeutic potential of miRNA-based interventions. miRNA mimics restore tumor-suppressive miRNAs (e.g., miR-125b, miR-34a) [209,213,214,215], while antagomirs, locked nucleic acids (LNAs), and miRNA sponges silence oncomiRs (e.g., miR-21, miR-138) [195,216,217]. Delivery strategies include lipid nanoparticles (LNPs), exosomes, chitosan/PLGA nanoplexes, and gold nanoparticles, which address challenges like nuclease degradation and bone marrow targeting (Table 3).

As summarized in Table 3, miRNA-based therapeutics for multiple myeloma remain exclusively preclinical as of the end of 2025, with no candidate having entered clinical testing in MM patients. The most translationally advanced miRNAs relevant to the field are miR-34a and miR-16 mimics, which are the only ones from Table 3 to have reached Phase I trials in other malignancies (MRX34 terminated in 2016 after immune-related serious adverse events; TargomiRs/mesomiR-16 completed Phase I in mesothelioma/NSCLC in 2017 with acceptable safety but no further development reported). Approaches using extracellular vesicles (EVs) or targeted nanoparticles/LNPs (e.g., miR-15a/16, miR-105-5p, miR-199a-5p, miR-221/222) have substantially higher translational potential than simple transfection-based methods because they begin to solve the critical barriers of systemic delivery, stability, and bone marrow homing that historically halted the entire miRNA therapeutic field. In contrast, the large majority of candidates (miR-21, miR-221/222 [transfection only], miR-19a, miR-25, miR-223, miR-27b-3p, miR-125b, miR-137, miR-138, miR-509-5p, etc.) are supported only by in vitro transfection studies and remain early-stage/conceptual for therapeutic application in patients.

Challenges include off-target effects, delivery efficiency, and the need for standardized protocols. Additionally, miRNAs like miR-105-5p, miR-744, and let-7e serve as prognostic biomarkers [218,219,220,221], offering potential for personalized therapeutic strategies [213,215,218]. Ongoing research aims to overcome these hurdles to translate miRNA therapeutics into effective MM treatments. Thus, miRNA mimics/antagomirs complement oligonucleotide strategies, but their full potential requires cross-modality comparison in delivery and clinical contexts, detailed below.

**Table 3:** Dysregulated miRNAs in MM.

miRNA	Function	RNA Platform	Effect	Delivery Method	Status (End of 2025)	Reference(s)
Anti-apoptotic						
miR-15a	Tumor suppressor, targets CABIN1	miRNA mimics	Promotes apoptosis, reduces proliferation	INTERFERin^®^ transfection	Preclinical, in vitro/murine	[222]
miR-16	Tumor suppressor, targets BCL2/NFkB	miRNA mimics	Promotes apoptosis, reduces proliferation	Lipid rafts (preclinical); EGFR-targeted bacterially derived minicells (EnGeneIC Dream Vector) in clinical candidate	Preclinical, in vitro/murine; clinically advanced miRNA mimic relevant to oncology—TargomiRs/mesomiR-16 (miR-16 mimic in EGFR-targeted minicells, EnGeneIC) completed Phase I in malignant pleural mesothelioma and NSCLC (NCT02369198, 2014–2017) with acceptable safety and disease stabilization signals, but no further development or MM trial as of 2025	[223]
Proliferation						
miR-34a	Tumor suppressor, targets AURKA	miRNA mimics	Inhibits proliferation, induces apoptosis	Chitosan/PLGA nanoplexes, nanogels (preclinical); liposomal formulation in clinical candidate	Preclinical, in vitro/murine; most translationally advanced miRNA mimic in oncology—MRX34 (liposomal miR-34a mimic, Mirna Therapeutics/Synlogic) reached Phase I in advanced solid tumors and hematologic malignancies (NCT01829971, 2013–2016) with some clinical activity signals but terminated due to immune-mediated serious adverse events (5 patient deaths); no resurrected miR-34a program or MM-specific trial as of 2025	[211,214]
miR-105-5p	Prognostic, regulates proliferation	miRNA mimics/inhibitors	Modulates survival (prognostic)	EV	Preclinical, exploratory	[221,224]
miR-19a	OncomiR, promotes proliferation	Antagomirs	Inhibits proliferation	Transfection (in vitro)	Preclinical, in vitro	[225,226]
miR-25	OncomiR, promotes proliferation	miRNA mimics	Inhibits proliferation	Transfection (in vitro)	Preclinical, in vitro	[227]
miR-223	OncomiR, promotes proliferation	Antagomirs	Inhibits proliferation	Transfection (in vitro)	Preclinical, in vitro	As a biomarker in MM [228], use of mimics in non-MM [229]
Drug Resistance						
miR-21	OncomiR, targets PTEN, promotes resistance	Antagomirs, LNAs	Enhances chemosensitivity	Transfection (in vitro)	Preclinical, in vitro	[230], miR-21 also can be nanoparticle encapsulated [231]
miR-221/222	OncomiR, promotes resistance	LNAs	Reduces progression, improves treatment	Transfection (in vitro)	Preclinical, in vitro	[232,233]
miR-106b-25	OncomiR, targets PCAF/p53	Antagomirs	Inhibits survival, enhances chemosensitivity	Transfection (in vitro)	Preclinical, in vitro	[234]
miR-181a/b	OncomiR, targets PCAF/p53	Antagomirs or mimics	Inhibits survival, enhances chemosensitivity	Transduction (in vitro)	Preclinical, in vitro	[235]
Microenvironment						
miR-27b-3p	OncomiR, promotes MM proliferation via FBXW7/c-MYC axis	miRNA sponges, LNAs	Inhibits proliferation, apoptosis resistance	Lipofectamine transfection (in vitro), LNP	Preclinical, in vitro	[236]
miR-214-3p	OncomiR, promotes fibroblast proliferation	miRNA sponges, LNAs	Inhibits proliferation, apoptosis resistance	Transfection (in vitro)	Preclinical, in vitro	[237]
miR-1343-3p	OncomiR, targets ATG7, suppresses autophagy	Antagomirs	Impairs survival, reduces autophagy	Lipofectamine transfection (in vitro)	Preclinical, in vitro	[194]
Angiogenesis						
miR-199a-5p	Tumor suppressor, targets HIF1A	miRNA mimics	Inhibits angiogenesis, MM progression	Nanoparticles or Lipofectamine	Preclinical, in vitro/murine	[238,239]
miR-10a	OncomiR, promotes proliferation via EPHA8 or IGF1R/CCND1/CUL3/ELAVL1	miRNA mimics	Inhibits proliferation	EVs	Preclinical, in vitro	[240,241,242]
miR-135b	OncomiR, promotes proliferation via activation of the Wnt/β-catenin-versican pathway and negative regulation of FIH-1 and SMAD5	miRNA mimics or inhibitors	Inhibits proliferation	EVs	Preclinical, in vitro	[240,243,244,245]
miR-346	OncomiR, promotes proliferation via βTRC	Antagomirs	Inhibits proliferation	EVs	Preclinical, in vitro	[240]
Genomic Instability						
miR-137	Tumor suppressor, targets AURKA	miRNA mimics	Reduces drug resistance, chromosomal instability	Transfection (in vitro)	Preclinical, in vitro	[246]
Immune Regulation						
miR-125b	Tumor suppressor, controls immune/apoptotic networks via inhibition of MKNK2, IRF4, PHLPP2 and MALAT1	miRNA mimics, LV vectors	Impairs growth, induces apoptosis	Transfection (in vitro)	Preclinical, in vitro/murine	[247,248,249,250]
miR-138	OncomiR, represses PAX5, TRPS1 and SULF2, promotes stemness	Antagomirs, LNA	Restores PAX5, reduces stemness	Transfection (in vitro)	Preclinical, in vitro	[197,210,212]
miR-509-5p	Tumor suppressor, regulates survival. Targets FOXP1 and MALAT1	miRNA mimics	Inhibits proliferation, enhances apoptosis	Transfection (in vitro)	Preclinical, exploratory	[162]
Prognostic (Exploratory)						
miR-15a	Prognostic, linked to survival. Targets CABIN1, VEGF-A and angiogenesis in MM	miRNA mimics, LNA	Modulates survival (prognostic)	EVs, LNPs	Preclinical, exploratory	[219,222,251,252,253,254]
miR-16	Prognostic, linked to survival. Targets CABIN1, the IKKα/β complex of the canonical NF-κB	miRNA mimics	Modulates survival (prognostic)	EVs, LNPs	Preclinical, exploratory	[222,223,227,254,255,256,257]
miR-744	Prognostic, linked to survival. Targets SOX12/Wnt/β-catenin pathway	miRNA mimics/inhibitors	Modulates survival (prognostic)	Transfection (in vitro)	Preclinical, exploratory	[258]
miR-92a	OncomiR, prognostic, linked to survival	miRNA mimics/inhibitors/LNA	Modulates survival (prognostic)	Transfection (in vitro)	Preclinical, exploratory	[259,260,261]
let-7e	Prognostic, linked to survival. Targets LIN28/let-7 axis regulating MYC.	miRNA mimics, LNA-GapmeR	Modulates survival (prognostic)	Transfection (in vitro)	Preclinical, exploratory	[218,262,263]
miR-221/222	Prognostic, tumor suppressor cluster; loss linked to progression and poor outcome	miRNA mimics	Modulates survival (prognostic), reduces quiescence/resistance	Transfection (in vitro), LNPs	Preclinical, exploratory	[262,264]
miR-665	OncomiR, prognostic; elevated in refractory MM, predicts poor OS/PFS	miRNA inhibitors	Modulates drug response/survival (prognostic)	Transfection (in vitro), EVs	Preclinical, exploratory	[265]
miR-193b-5p/miR-483-3p/let-7b-5p	Prognostic panel from exosomal miRNA; low expression predicts poor OS	miRNA mimics (for tumor suppressors)	Modulates survival/prognosis; diagnostic panel AUC 0.94	EVs, LNPs	Preclinical, exploratory	[266,267]

EVs, Extracellular Vesicles; LNPs, Lipid Nanoparticles.

#### 3.1.3. Long Non-Coding RNAs (lncRNAs) as Targets in MM

Long non-coding RNAs (lncRNAs), defined as transcripts longer than 200 nucleotides that lack protein-coding potential, regulate key cellular processes such as transcription, splicing, chromatin remodeling, and RNA stability. Dysregulated lncRNAs are frequently implicated in the pathogenesis, progression, and drug resistance of multiple myeloma (MM), making them compelling candidates for RNA-based therapeutic intervention.

Recent studies have highlighted multiple oncogenic and tumor-suppressive lncRNAs in MM. Among these, MALAT1, HOTAIR, and RP11-350G8.5 are most prominently associated with disease progression, immune evasion, bone lesions, and resistance to therapy (Table 4) [9,20,155,156,179]. The role of exosomal lncRNAs, such as NEAT1 and SNHG16, in modulating the tumor microenvironment and enhancing chemoresistance further supports the potential of lncRNAs as both biomarkers and therapeutic targets [20,179].

MALAT1

The metastasis-associated lung adenocarcinoma transcript 1 (MALAT1) is one of the most studied lncRNAs in MM. MALAT1 is overexpressed in advanced MM and correlates with extramedullary disease, chemoresistance, and poor prognosis [157,158,164]. It promotes MM survival under hypoxia by regulating the HIF-1α-KDM3A axis [159], and interacts with the poly(A) polymerase FAM46C, a known tumor suppressor, enhancing cell migration and invasion [161].

MALAT1 also functions as a competing endogenous RNA (ceRNA), sponging tumor-suppressive miRNAs such as miR-125b, miR-1271-5p, and others, thereby promoting oncogenic pathways [162,163,169,250,274]. Studies from 2025 further link MALAT1 to lenalidomide resistance through CD38 epigenetic modulation and oxidative stress, with ASO targeting restoring sensitivity in models [165].

Therapeutic targeting of MALAT1 can be achieved with ASOs (e.g., FTX-001) and locked nucleic acid (LNA) gapmeRs targeting MALAT1, which have shown potent efficacy in preclinical models [166,167,168]. Additionally, combination therapies with PARP1 inhibitors or bortezomib synergistically reduce MM viability [167,168] (see Supplementary Tables S1 and S2, which comprehensively summarize MALAT1’s oncogenic functions, biomarker correlations, preclinical targeting evidence—including the clinical-stage ASO FTX-001—and patient-derived data directly supporting its central role in proliferation, chemoresistance, hypoxia adaptation, and microenvironment remodeling in MM).

HOTAIR

The lncRNA HOTAIR is similarly upregulated in MM, particularly in aggressive and bone-invasive disease, and is associated with glucocorticoid resistance [171,268]. It promotes NF-κB and JAK2/STAT3 pathway activation, driving proliferation and survival, and interfering with apoptosis [152,153]. Knockdown of HOTAIR using ASOs or siRNAs restores dexamethasone sensitivity, promotes apoptosis, and inhibits MM cell migration and invasion [171,269]. Elevated HOTAIR levels are also observed in MM patients with bisphosphonate-induced osteonecrosis of the jaw (BRONJ), linking HOTAIR to bone pathology [172] (see Supplementary Tables S3 and S4, which detail HOTAIR’s mechanisms in NF-κB/JAK-STAT activation, glucocorticoid resistance, bone pathology, and exosomal transfer, together with key experimental and clinical correlations that reinforce its therapeutic and prognostic relevance).

RP11-350G8.5

The lncRNA RP11-350G8.5 contributes to MM proliferation, drug resistance, and relapse. Its expression is upregulated in relapsed/refractory MM and correlates with poor outcomes [176,177,180]. Silencing RP11-350G8.5 using RNAi approaches in vitro significantly inhibits MM growth [176]. CRISPR-Cas9 screens in 2024–2025 validate RP11-350G8.5 as an essential lncRNA for MM fitness, with LNA ASOs showing therapeutic potential in reducing cell viability [176].

FAM46C

Loss of the tumor suppressor FAM46C, frequently mutated in MM, is associated with increased MALAT1 expression and is linked to enhanced MM cell migration and survival [154]. Disruption of this axis is an emerging therapeutic strategy, particularly in cases with FAM46C loss-of-function mutations. Studies from 2025 show FAM46C expression sensitizes MM cells to PF-543 cytotoxicity, highlighting its role in sphingolipid metabolism and potential synergy with RNA-based MALAT1 targeting [275], (see Supplementary Tables S1–S4).

Targeting lncRNAs like MALAT1 via ASO/siRNA extends RNA silencing modalities, warranting comparative evaluation with mRNA-based approaches in delivery and therapeutic synergy.

### 3.2. mRNA-Based Cell Engineering Platforms

The advent of messenger RNA (mRNA)-based platforms has revolutionized adoptive immunotherapy for multiple myeloma (MM) by enabling transient, non-integrative expression of chimeric antigen receptors (CARs) in immune effector cells. Unlike viral vector-based approaches, mRNA engineering offers controllable, repeatable dosing with reduced toxicity, eliminating the need for lymphodepleting chemotherapy and minimizing risks like cytokine release syndrome (CRS) and neurotoxicity. While early platforms focused on ex vivo engineering, 2024–2025 advancements include in vivo mRNA delivery via lipid nanoparticles (LNPs) for scalable, off-the-shelf applications, potentially reducing manufacturing costs. This section explores mRNA-engineered CAR-T cells, NK cells, γδ T cells, and emerging in vivo programming platforms, highlighting their preclinical and clinical outcomes, mechanisms, and potential as off-the-shelf or personalized therapies for MM.

#### 3.2.1. Descartes-08 and Descartes-11: Transient mRNA CAR-T Platforms

Descartes-08, developed by Cartesian Therapeutics, is a pioneering mRNA-engineered CAR-T therapy targeting B-cell maturation antigen (BCMA) in autologous CD8^+^ T cells. Using codon-optimized synthetic mRNA electroporated ex vivo, Descartes-08 expresses a second-generation CAR with CD28 and CD3ζ signaling domains, flanked by mouse alpha-globin 5′ and 3′ UTRs for enhanced translation and stability [188]. The transient CAR expression (~5–7 days) allows weekly outpatient infusions without lymphodepletion, reducing CRS risk. This is in line with in vivo preclinical studies using nanoparticle-delivered mRNA in mouse models demonstrating transient CAR expression peaking at day 2 and downregulating by day 7 [276]. In vitro, Descartes-08 achieved 66–82% lysis of CD138^+^ primary MM cells and robust cytotoxicity against MM cell lines (MM1S, RPMI-8226, H929), including lenalidomide- and pomalidomide-resistant variants, with strong IFN-γ, TNF-α, and IL-2 secretion (Table 5). In MM1S-luc xenograft models, weekly dosing extended survival to 69 days versus 43–44 days in controls (*p* < 0.0001) [277]. Clinically, a phase I trial (NCT03448978, terminated after completion) reported a stringent complete remission (sCR) in a relapsed/refractory MM (RRMM) patient with plasma cell leukemia after three doses, with no adverse events (AEs); Table 6. However, further MM development was halted, with focus shifting to autoimmune indications. As of 2025, Cartesian has advanced Descartes-15, a next-generation autologous anti-BCMA mRNA CAR-T, into Phase 1 for RRMM (first patient dosed 2024), emphasizing outpatient safety without lymphodepletion. Both therapies are also being repurposed for autoimmune diseases, such as myasthenia gravis (NCT04146051, NCT06799247) [278] and systemic lupus erythematosus (NCT06038474), leveraging their transient expression for safety.

Despite the strategic deprioritization of oncology programs in 2024–2025, mRNA-engineered autologous anti-BCMA CAR-T cells from Cartesian Therapeutics (Descartes-08, Descartes-11, and especially the next-generation Descartes-15) remain the most clinically mature RNA-based therapeutic modality ever tested in multiple myeloma patients—and by a wide margin the closest to potential therapeutic utility among all RNA approaches reviewed here. These are the only RNA therapeutics that have treated substantive numbers of MM patients in the clinic (>35 patients across all trials as of December 2025), achieved multiple stringent complete responses with MRD negativity in both relapsed/refractory and high-risk consolidation settings, and demonstrated an unprecedented safety profile for a cellular therapy (fully outpatient administration, no lymphodepleting chemotherapy, no CRS or ICANS of any grade). The Phase 1 study of Descartes-15 (NCT06304636), which incorporated mRNA optimizations yielding ~10-fold higher and more persistent CAR expression than Descartes-08/11, completed its dose-escalation portion in November 2025 with similarly excellent tolerability before the program was paused on 13 November 2025, to reallocate resources to autoimmune indications.

The company’s strategic pivot away from oncology underscores the fundamentally different therapeutic objectives and disease biology between multiple myeloma and autoimmune diseases. In autoimmune conditions, the primary goal is immunomodulation and restoration of tolerance, not complete cellular eradication. Transient CAR-mediated depletion of autoreactive B cells or plasma cells is, therefore, not only sufficient but ideal—it can achieve profound clinical responses by resetting the pathogenic immune repertoire while prioritizing safety and avoiding the long-term risks of persistent CAR-T activity (e.g., secondary malignancies, prolonged cytopenias). In multiple myeloma, by contrast, the therapeutic aim is complete and durable elimination of a genetically unstable, heterogeneous, and adaptative malignant clone. The clonal heterogeneity, genomic instability, and high propensity for antigen escape (e.g., BCMA downregulation) intrinsic to myeloma are not merely challenges of persistence, but fundamental drivers of relapse that demand a more sustained and potent effector response. Coupled with the protective bone marrow niche, these factors create a disease that requires prolonged immunosurveillance and/or repeated interventions to target evolving subclones and prevent resurgence—a rationale for the lifelong engraftment strategy of approved viral CAR-T products, despite their associated toxicities.

Cartesian’s entirely unarmored, purely transient platform, while brilliantly safe, may, therefore, be biologically underpowered for the unique demands of the MM bone marrow niche without additional engineering. No Cartesian product to date has incorporated armored cytokines (e.g., membrane-bound IL-15, secreted IL-12, or IL-18) or other enhancements—all clinical candidates have been simple second-generation anti-BCMA CARs. Future iterations (should the company or others revisit oncology) could dramatically improve performance through straightforward mRNA modifications already validated preclinically elsewhere:

- Multi-cistronic mRNA co-expressing the CAR with membrane-tethered IL-15 or IL-15/IL-15Rα fusion for enhanced persistence and memory phenotype in the hypoxic, TGF-β-rich bone marrow microenvironment;
- Additional cytokine modules (e.g., IL-18 or IL-12) to resist T-cell exhaustion and recruit innate immunity;
- Chemokine receptors (e.g., CXCR4, CCR7) or bone marrow-homing domains to improve trafficking and retention in the endosteal niche;
- Dual- or trispecific CAR constructs targeting BCMA + GPRC5D ± CD38 or other combinations to preempt antigen escape;
- Transition to circular RNA (circRNA) backbones, which would extend functional persistence from days to weeks while preserving the non-integrative safety profile.

Such armored, niche-adapted transient mRNA CAR-T platforms could in principle combine the best attributes of current viral CAR-T (deep, durable responses) with Cartesian’s unparalleled safety, making them ideally suited for earlier-line use, consolidation therapy, or sequencing with other modalities in MM. Until such enhancements are implemented, however, the transient mRNA CAR-T modality—despite being the most clinically advanced RNA therapy in multiple myeloma history—remains paused in oncology.

#### 3.2.2. mRNA-Engineered CAR-NK and CAR-γδ T Cells

mRNA engineering extends beyond T cells to non-conventional effectors like NK cells and γδ T cells, which offer innate tumor recognition and reduced graft-versus-host disease (GVHD) risk, making them ideal for allogeneic, off-the-shelf therapies (Table 7). Lipid nanoparticle (LNP)-based mRNA delivery has generated BCMA-CAR NK cells with potent cytotoxicity against MM1S and RPMI-8226 cells, accompanied by robust IFN-γ and Granzyme B secretion [279]. Dual mRNA constructs co-expressing BCMA-CAR and CXCR4 enhance bone marrow homing, improving tumor control in MM xenografts [280]. Similarly, Vγ9Vδ2 γδ T cells electroporated with BCMA-CAR mRNA demonstrated tumor regression and prolonged survival in KMS-11-luc xenograft models [281]. Dual CAR (CD19^+^BCMA) NK-92 cell lines, engineered via mRNA electroporation, showed synergistic cytotoxicity across B-cell and plasma cell malignancies, mitigating antigen escape [124]. Additionally, CRISPR-mediated CD38 knockout combined with CD38-CAR mRNA in NAM-cultured NK cells produced fratricide-resistant effectors with enhanced lysis of CD38^+^ MM cells [282]. These platforms highlight the versatility of mRNA engineering for scalable, allogeneic MM therapies. Recent 2024–2025 updates include IL-12 co-expression in mRNA CAR-NK for amplified cytotoxicity against MM, with safe incorporation in preclinical models. While promising, no Phase I MM trials for mRNA CAR-NK/γδ T as of the end of 2025; focus remains on optimizing persistence.

As summarized in Table 7 and Table 8, all mRNA-engineered NK, γδ T, in vivo CAR programming, and alternative cell platforms remain strictly preclinical as of December 2025, with no candidates having entered clinical testing in multiple myeloma or any other indication. These approaches show strong conceptual promise—particularly for bone marrow homing (e.g., CXCR4 co-expression), fratricide resistance, and true off-the-shelf scalability—but lag far behind the clinical maturity of the autologous mRNA CAR-T platforms (Descartes-08, Descartes-11, and Descartes-15), which are the only RNA-based cell therapies to have reached the clinic in multiple myeloma, treated >35 patients across trials, and achieved multiple stringent complete responses with MRD negativity.

#### 3.2.3. In Vivo mRNA-Based CAR-T Programming

A novel frontier in MM immunotherapy involves in vivo programming of T cells using antigen-presenting nanoparticles (APNs) functionalized with peptide–MHC complexes. These APNs selectively transfect antigen-specific T cells with BCMA-CAR mRNA, bypassing ex vivo manipulation [284]. In U266-luc NSG mouse models, APN-mediated delivery achieved CAR expression comparable to lentiviral CAR-T, with significant tumor control and minimal off-target effects due to T-cell receptor (TCR) restriction. This scalable, minimally invasive platform represents a paradigm shift toward off-the-shelf, non-viral CAR-T therapies for MM, with ongoing optimization for human translation in 2025. Moreover, combining mRNA therapies with immunomodulatory drugs (e.g., lenalidomide) could enhance efficacy, as seen in preclinical models [277]. Emerging strategies may enhance scalability.

#### 3.2.4. Challenges and Future Directions for mRNA-Based Cell Engineering Approaches in MM

Despite their promise, mRNA-engineered cell therapies face challenges, including transient expression requiring repeated dosing, potential off-target effects, and the need for optimized delivery to the bone marrow microenvironment [278,279,281,283]. The lack of MM-specific clinical trials beyond early-phase studies (e.g., NCT03448978, NCT04436029) underscores the need for further validation (Table 8). Emerging strategies, such as selective organ targeting (SORT) LNPs for in vivo T-cell delivery [286,287], are in preclinical exploration and may enhance scalability and accessibility. In parallel, immunomodulatory drugs (e.g., lenalidomide) in combination with mRNA therapies might show better efficacy, confirmed by preclinical models [277].

While the focus has often been on T-cells or other traditional cytotoxic cells as carriers, mRNA modalities can also be combined with alternative cell types for best results. For example, early trials (e.g., NCT01995708, Phase I completed) used mRNA-electroporated autologous Langerhans DCs to induce anti-MM immunity post-autotransplant, showing safety and immunogenicity but limited efficacy data [285].

#### 3.2.5. Take-Home Summary for mRNA-Based Approaches in MM

mRNA-based CAR engineering offers transient, controllable, and safe immunotherapy options for MM, with platforms like Descartes-08 and Descartes-11 demonstrating preclinical and early clinical efficacy. However, MM development for Descartes-08 and Descartes-11 paused, with successors like Descartes-15 advancing in 2025. Extending mRNA engineering to NK and γδ T cells provides allogeneic, off-the-shelf solutions, while in vivo APN platforms promise scalable, non-invasive CAR-T generation. Despite challenges like transient expression and delivery optimization, mRNA therapies represent a transformative approach to MM treatment, with potential synergies when combined with existing therapies and novel mRNA vaccines. mRNA-engineered cells thus address key barriers, but their advantages become evident through comparative assessment with vaccine and delivery platforms in subsequent analyses. Complementing these cell-engineering approaches, a BCMA-mRNA vaccine has shown therapeutic potential in MM models by eliciting anti-BCMA immunity without requiring cell manipulation [147], a strategy further detailed in the following section on non-cell-based mRNA modalities.

### 3.3. mRNA Vaccines in MM

#### 3.3.1. mRNA Vaccine Responsiveness in MM

Patients with multiple myeloma (MM) exhibit profound immune dysregulation, characterized by compromised B-cell and T-cell function, which can impair responsiveness to mRNA-based therapies, including vaccines. This has been particularly evident in the context of anti-SARS-CoV-2 vaccination. Several studies report suboptimal immunogenicity in MM patients, with seroconversion rates ranging from 76.9% to 88.2% and significantly diminished T cell-mediated responses (~36.2%) after mRNA vaccination. Underlying contributors include active disease, elevated bone marrow plasmacytosis, immunosuppressive treatment regimens, and anemia [34,288,289,290,291].

These findings raise concerns about the readiness of MM patients to benefit from mRNA-based therapeutic strategies, including vaccines encoding MM-associated antigens. Notably, a third vaccine dose boosts antibody responses in most MM patients (~84%), though initial responses are frequently impaired and short-lived, with titers declining substantially within 6 months [289,290]. Data from 2025 from ESMO and meta-analyses confirm improved antibody positivity (90–95%) with bivalent boosters, though neutralizing titers remain 2–5× lower than healthy controls, and T-cell responses hover at 40–50% [292]. Persistent vulnerabilities underscore the need for repeated dosing or adjuvants to sustain immunity [291].

mRNA vaccines rely on intracellular translation and MHC class I presentation, processes that may be hindered in the immunosuppressive environment of MM. Therefore, immunomodulatory strategies—such as combination with immune adjuvants, immune checkpoint inhibitors, or cytokine support—may be essential to augment responses [293,294,295].

Moreover, emerging reports emphasize the need for precision-based immunization strategies tailored to individual MM disease status and treatment exposure [296,297,298,299,300].

#### 3.3.2. mRNA Vaccines for MM: Preclinical and Clinical Insights

mRNA vaccines are emerging as a novel immunotherapeutic strategy in multiple myeloma (MM), aiming to elicit tumor-specific immune responses through the delivery of tumor-associated antigens (TAAs) and neoantigens (Table 9). As summarized in Table 9, true antigen-encoding mRNA vaccines remain exclusively preclinical in multiple myeloma as of December 2025. Among them, the BCMA + poly(I:C) LNP vaccine is the most translationally advanced and the only candidate realistically close to clinical testing, with its 2025 full publication in Blood explicitly positioning it for Phase 1 evaluation due to outstanding preclinical efficacy and safety [147]. All other mRNA vaccine approaches in MM remain early-stage or conceptual. Two early-phase clinical trials have explored this approach. NCT00965224 employed dendritic cells (DCs) electroporated with WT1 mRNA in patients with acute myeloid leukemia (AML) and included MM patients in its exploratory scope [300]. The trial NCT00965224 demonstrated that WT1 mRNA-electroporated DCs could prevent or delay relapse in 43% of high-risk AML patients and improved 5-year overall survival (OS) compared to population registry data. Clinical response was correlated with WT1-specific CD8^+^ T-cell expansion and cytokine production [301]. NCT01995708 evaluated post-autologous stem cell transplant vaccination using Langerhans-type DCs electroporated with CT7, MAGE-A3, and WT1 mRNAs in MM patients. [285]. The vaccines were well tolerated and induced antigen-specific CD4^+^ and CD8^+^ T-cell responses, with increased pro-inflammatory cytokine secretion and cytotoxic marker expression. Although the study was not powered for efficacy, treatment responses favored the vaccine arm [285].

A 2023 ASH abstract reported the development of a lipid nanoparticle (LNP)-based mRNA vaccine encoding BCMA, combined with the adjuvant Poly I:C to enhance immunogenicity. The vaccine induced robust antigen expression in dendritic cells and enhanced CD8^+^ T cell activation both in vitro and in vivo. In co-culture assays, BCMA + Poly I:C LNPs achieved >70% CFSE dilution in naïve T cells, and the resulting cytotoxic T lymphocytes (CTLs) selectively lysed BCMA^+^ MM cells. In C57BL/6 mice, splenic accumulation of BCMA LNPs led to DC activation (CD40^+^/CD80^+^), increased BCMA-specific CD8^+^ T cells (tetramer+), and effective lysis of syngeneic 5TGM1 MM cells. Poly I:C further amplified T cell infiltration (CD3^+^, CD8^+^) and cytokine production (IFNγ, TNFα). Biodistribution, safety (H&E), and IF staining confirmed the vaccine’s tolerability and immunostimulatory efficacy, highlighting its translational potential for therapeutic vaccination in MM [295]. A 2025 publication of this work confirms preclinical efficacy, with no CRS in models, positioning it for Phase 1 transition [147].

Broader reviews emphasize the feasibility of targeting splicing-derived neoantigens such as RHAMM [302], and the promise of AI-enhanced LNP platforms in refining antigen design clinical [303,304,305]. Notably, a first-in-human clinical trial (NCT03631043) is currently evaluating a personalized peptide-based neoantigen vaccine in patients with smoldering multiple myeloma, aiming to delay progression to overt MM [305]. Although details remain limited and not yet peer-reviewed as of December 2025, Moderna’s mRNA-2808 (Phase 1, NCT07116616, first patient dosed November 2025 in relapsed/refractory MM) is widely understood to be an intravenously administered mRNA-LNP encoding a multispecific (e.g., potentially tri-specific) T-cell engager rather than a traditional antigen-encoding therapeutic vaccine. Safety, tolerability, and pharmacokinetic learnings from this first-in-human oncology mRNA-LNP trial will nonetheless be highly relevant for future mRNA-based therapeutics in multiple myeloma, including eventual therapeutic vaccine platforms. However, no mRNA-based neoantigen vaccines specific to MM have yet entered clinical trials. These findings collectively highlight the translational potential of mRNA vaccines in MM, while also underscoring the need for continued optimization of antigen selection, delivery systems, and immune adjuvants for clinical efficacy.

Thus, while clinical experience is currently limited to old DC-based trials and no LNP-mRNA vaccine has reached the clinic in MM, the BCMA + poly(I:C) platform stands out as the clear leader with the highest near-term translational potential.

**Table 9:** mRNA-based therapeutic vaccines in multiple myeloma—translational status.

Approach	Antigen(s)	Platform	Preclinical Model(s)	Key Outcomes	Status (as of End of 2025)	Reference(s)
WT1 mRNA-electroporated DCs	WT1	Autologous DCs + mRNA electroporation	AML (main), limited MM patients	Antigen-specific T-cell responses; relapse prevention in subset	Phase 2 completed (NCT00965224, exploratory MM cohort); no further development in MM	[301]
CT7/MAGE-A3/WT1 mRNA-electroporated LCs	CT7, MAGE-A3, WT1	Langerhans-type DCs + mRNA electroporation	Post-ASCT MM	Safe; induced CD4^+^/CD8^+^ responses; trend toward better treatment responses	Phase 1 completed (NCT01995708); no follow-up trials	[285]
BCMA + Poly I:C LNP vaccine	BCMA	Ionizable LNP (lipid 5) + poly(I:C) adjuvant	Human MoDCs, U266, patient BM; syngeneic 5TGM1 (murine)	Robust DC activation, BCMA-specific CD8^+^ CTLs, selective MM lysis, tumor control; excellent safety	Strictly preclinical; most translationally advanced true mRNA vaccine in MM (explicitly positioned for Phase 1 transition in 2025 publication)—realistically closest to clinical testing	[147,295]
Galsomes BCMA neoepitope vaccine	BCMA neoepitopes	Galsomes mRNA-LNP	MM xenografts	CD8^+^/iNKT activation, superior to traditional vaccines	Strictly preclinical/early-stage	[306]
RHAMM splicing neoantigen concepts	RHAMM, UBR4, PRKDC, mtDNA SNPs	Conceptual/AI-predicted mRNA-LNP	In silico + limited in vitro	High immunogenic potential identified	Conceptual/early preclinical—no vaccine constructed yet	[302,303,304,305]

#### 3.3.3. Neoantigen Discovery and mRNA Vaccine Potential in MM

Recent developments in next-generation sequencing (NGS) and artificial intelligence (AI)-assisted epitope prediction have broadened the identification of neoantigens specific for MM. This paved the way for tailored mRNA vaccine strategies (Table 9). These neoantigens arise from somatic mutations (e.g., SNVs, INDELs), aberrant splicing, and mitochondrial DNA mutations, offering individualized therapeutic targets. For instance, aberrant splicing of RHAMM, combined with common mutations in UBR4 and PRKDC, have been identified as immunogenic across MM progression phases. Mitochondrial SNPs, due to their elevated mutation frequency, further enrich the pool of potential neoantigens, as evidenced by recent experimental models. AI-powered tools, such as NetMHCpan, DeepVACPred, and AlphaFold 3, have significantly improved precisions of HLA class I neoepitopes, supporting more accurate vaccine design. Immunopeptidomics research from 2025 studies highlight Galsomes mRNA-LNP vaccines aimed at BCMA neoepitopes, activating CD8^+^/iNKT cells, outperforming traditional vaccines in MM models [306]. A 2024 ASH presentation demonstrated the potential of LNP-formulated mRNA vaccines against BCMA neoepitopes, generating robust T-cell activation responses in MM xenograft models [147,295]. While early in clinical stages, peptide-based personalized neoantigen vaccines are being explored in smoldering MM (NCT03631043). These initiatives highlight the translational potential of neoantigen-based mRNA vaccines in MM, even as dedicated MM-specific mRNA clinical trials are yet to emerge. mRNA-based therapies have demonstrated potential in other hematological malignancies, such as AML, where they can elicit potent immune responses. For example, in a phase 2 trial (NCT00965224), WT1 mRNA-electroporated dendritic cells in high-risk AML patients induced antileukemic responses in 13 of 30 cases, with improved 5-year OS in responders (53.8% vs. 25.0%; *p* = 0.01) linked to WT1-specific CD8^+^ T-cells—suggesting similar mRNA-DC platforms targeting tumor antigens could be adapted for MM [301] (Table 9).

Multiple myeloma poses uniquely severe challenges for neoantigen-based vaccination that are substantially greater than in high-TMB solid tumors: extremely low mutational burden (typically yielding only 5–20 candidate neoepitopes per patient), extensive subclonal heterogeneity, rapid antigen loss/escape, and the profoundly immunosuppressive bone marrow niche (high TGF-β, IL-10, MDSCs, Tregs, and dysfunctional/exhausted dendritic cells). These factors combine to produce poor antigen presentation, weak T-cell priming, and rapidly waning responses—barriers already clinically validated by the impaired and short-lived anti-SARS-CoV-2 mRNA vaccine responses in MM patients (Section 3.3.1).

Successful translation will, therefore, require deliberate combination strategies now actively pursued in 2025–2026 preclinical and early-clinical programs:

- Prioritizing non-canonical, clonally dominant neoantigens (RHAMM aberrant splicing isoforms, UBR4/PRKDC recurrent mutations, mitochondrial SNPs) that are more consistently expressed across MM stages than sparse SNV/INDEL-derived epitopes [302,303,304,305]
- Multi-antigen cocktails combining private neoantigens with shared TAAs (BCMA, CD38, CS1) or myeloma-specific idiotypic determinants
- Mandatory partnering with niche-opening therapies (anti-CD38 mAbs, IMiDs, BCMA-targeted ADCs/BiTEs/CAR-T) administered immediately before or during vaccination to transiently deplete immunosuppressive plasma cells and improve T-cell trafficking
- Bone marrow-targeted delivery systems (SORT-LNPs, CXCR4- or CD138-conjugated nanoparticles) and potent built-in adjuvants (STING agonists, TLR7/8 ligands, IL-12 mRNA)
- Self-amplifying or circular RNA backbones for prolonged antigen expression and stronger memory formation

The ongoing iVAC-XSAGA personalized peptide neoantigen vaccine trial in high-risk smoldering multiple myeloma (NCT03631043, still recruiting with preliminary immunogenicity data anticipated in 2026) and the strong preclinical foundation of the BCMA + poly(I:C) LNP vaccine—whose authors explicitly conclude that “analogous mRNA vaccination against immunogenic neoantigens determined by sequencing samples from patients with MM may lead to the development of personalized vaccines in MM”—together provide the clearest near-term opportunities to validate these multimodal strategies in patients. The peptide-based multi-epitope cocktail tested in iVAC-XSAGA can be directly transferred into the same or similar mRNA-LNP platforms (with or without poly(I:C)), enabling head-to-head comparison of shared-antigen (BCMA) versus personalized neoantigen approaches and likely superior efficacy of the latter due to better MHC-I presentation, broader clonal coverage, and reduced risk of antigen-loss escape. mRNA vaccines’ adaptability positions them as a high-potential modality, with comparative insights on delivery challenges and clinical sequencing provided in the following sections.

### 3.4. Comparative Analysis of RNA Delivery Systems in MM

Building on the RNA modalities discussed, this comparative analysis evaluates delivery systems’ ability to overcome MM-specific barriers, highlighting synergies across platforms for enhanced therapeutic outcomes. RNA-based therapeutics, including messenger RNA (mRNA), small interfering RNA (siRNA), antisense oligonucleotides (ASOs), and microRNA (miRNA) mimics or inhibitors, have emerged as promising modalities for the treatment of multiple myeloma (MM). These approaches enable precise modulation of oncogenic and immunoregulatory pathways such as B-cell maturation antigen (BCMA), MYC, and cytokine signaling. Despite their potential, efficient delivery to the bone marrow microenvironment—where MM cells predominantly reside—remains a major obstacle. Challenges include limited endosomal escape, off-target effects, and rapid immune clearance.

**Figure 5:**
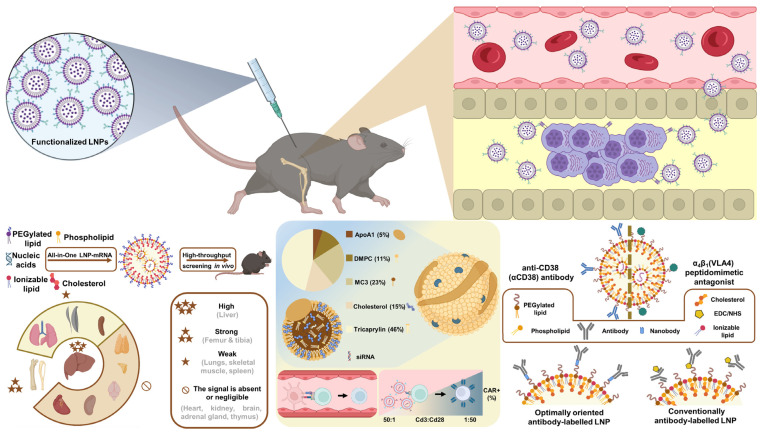
Functionalized lipid nanoparticles (LNPs) for targeted RNA delivery in multiple myeloma (MM): overcoming bone marrow (BM) niche challenges and bio-inspired strategies. This schematic illustrates LNP design for BM-targeted RNA therapy in MM, addressing niche barriers (e.g., fibrosis, hypoxia) and incorporating antibody orientation and co-stimulation. (**Top Center and Top Right**): BM niche challenges include TGF-β-driven fibrosis (collagen I/III crosslinking), hypoxia (pO_2_ < 5 mmHg), dense ECM (hyaluronan/heparan sulfate), high interstitial pressure, MM cell efflux pumps, and macrophage/MDSC scavenging. To reach this niche, antibody decoration (e.g., anti-CD38 [5]) or naturally inspired ApoA1 (enhances BM tropism [310]) can be used. (**Top and Bottom Left**): Functionalized LNPs with PEGylated lipid (stealth/stability), phospholipid (disperses aggregates), nucleic acid, ionizable lipid (endosomal escape), and cholesterol (structural integrity). Encapsulates RNA (e.g., siRNA) for intravenous injection into MM mouse models, as in αCD38-tLNP siRNA-CKAP5 studies ([5]; IV for systemic BM delivery). (**Bottom Center**): ApoA-coated LNP composition, DMPC (phospholipid: stabilizes with ApoA1), cholesterol (structural organization), tricaprylin (promotes spherical stability), MC3 (ionizable lipid for RNA complexation), ApoA1 (BM targeting/stability) [309]. Antibody ratios (e.g., CD3/CD28 on aLNPs) affect cargo expression (e.g., higher CD28 ratios increase CAR+% in T-cells via bio-inspired co-stimulation [312]). (**Bottom Right**): Antibody orientation is critical; nanobody-mediated (e.g., anti-Fc nanobody) ensures site-specific conjugation and antibody orientation, improving LNP targeting [311]. Abbreviations: aLNPs, activating antibody-labeled lipid nanoparticles; ApoA1, apolipoprotein A1; BM, bone marrow; CAR, chimeric antigen receptor; CD, cluster of differentiation; DMPC, dimyristoylphosphatidylcholine; ECM, extracellular matrix; EDC, 1-ethyl-3-(3-dimethylaminopropyl)carbodiimide; IV, intravenous; LNP, lipid nanoparticle; MC3, DLin-MC3-DMA; MDSC, myeloid-derived suppressor cell; MM, multiple myeloma; mRNA, messenger RNA; NHS, *N*-hydroxysuccinimide; PEG, polyethylene glycol; siRNA, small interfering RNA; TGF-β, transforming growth factor beta; tLNP, targeted lipid nanoparticle; VLA-4, very late antigen-4.

Recent advances (2023–2025) highlight the development of non-viral nanocarriers designed to enhance tissue-specific delivery, molecular stability, and biocompatibility while minimizing systemic toxicity. These innovations, demonstrated in preclinical studies and early-phase clinical investigations, underscore a growing trend toward the integration of engineered lipid nanoparticles, polymeric systems, exosome-based platforms, and ligand-decorated platforms for targeted RNA delivery in MM. The following section summarizes the latest progress across these RNA modalities, integrating findings from recent peer-reviewed publications, conference presentations, and translational research reports [307,308] (Figure 5).

#### 3.4.1. Lipid Nanoparticles (LNPs)

Lipid nanoparticles (LNPs) represent the most clinically advanced non-viral platform for RNA delivery. Their success stems from the use of ionizable lipids that facilitate efficient RNA encapsulation, endosomal escape, and controlled release into the cytoplasm [309]. In multiple myeloma (MM), targeted LNP formulations have demonstrated improved bone marrow tropism and reduced hepatic accumulation, addressing key barriers in systemic RNA delivery [5,310].

Recent studies (2023–2025) have expanded the potential of LNPs for both gene silencing and immune modulation in MM. Preclinical data indicate that CD38-targeted LNPs can achieve efficient knockdown of oncogenic transcripts in MM cells while sparing healthy hematopoietic populations [5,307]. Complementary research has introduced optimized ionizable lipid designs capable of co-delivering siRNA and ASOs with enhanced bone marrow penetration [310]. Another innovative platform employs antigen-presenting cell-mimetic LNPs for selective mRNA transfer into T cells, achieving precise in vivo targeting with a potential of tumor reduction in MM xenograft models [311,312]. Furthermore, methodological advances have refined LNP architecture to improve mRNA stability and immunocompatibility, highlighting rational design strategies for hematologic malignancies [5,313,314,315,316,317,318,319] (Figure 5).

Conference reports from ASH 2024 and EHA 2025 underscored this trend, describing LNP-mediated mRNA approaches for BCMA-targeted therapies [143,320] and MM-specific mRNA vaccines [306]. Notably, ionizable lipid innovations achieved measurable response rates in relapsed/refractory MM (RRMM) preclinical models, while immunopeptidomic profiling identified neoantigens suitable for α-galactosylceramide–adjuvanted formulations (e.g., Galsomes) [306].

From a translational perspective, early-phase clinical development continues to advance. In 2024, an mRNA-engineered anti-BCMA CAR-T candidate, Descartes-15, entered Phase I evaluation for relapsed/refractory multiple myeloma (RRMM) (NCT06304636) [321,322], which achieved durable responses without lymphodepleting conditioning. While most of Cartesian Therapeutics’ publicly described platforms involve serial administration of mRNA-electroporated CAR-T cells, their patent WO2024197098 outlines a complementary approach employing lipid nanoparticles (LNPs) for direct intra-lymph node delivery of CAR-encoding mRNA to enable in situ T-cell transfection. Within this patent, apolipoprotein E (ApoE) is explicitly described as a transfection agent that binds to the LNP surface, enhancing cellular uptake, endosomal escape, and mRNA expression in lymph node–resident immune cells while minimizing systemic exposure. This localized strategy contrasts with systemic ApoE-dependent liver or bone marrow tropism reported elsewhere [5,310,323,324,325], underscoring its intended use for sustained local transfection and in vivo CAR-T generation in lymphoid tissues.

Beyond lymph node-restricted approaches, other preclinical efforts have demonstrated that VLA-4-targeted LDV-functionalized LNPs can significantly enhance bone marrow accumulation and siRNA delivery by promoting interaction with resident leukocytes, providing proof of concept for targeted RNA therapeutics in hematologic malignancies [326]. Most recently reported rational lipid design has enabled the development of structurally optimized LNPs capable of targeting the bone marrow in an antibody-independent manner [325].

Together, these innovations indicate that LNP continue to dominate due to their scalability and highlight the adaptability of LNP systems for immune-cell-directed RNA delivery and with potential for lymph node or bone marrow targeting crucial for MM therapy through precise, modular, and scalable design.

#### 3.4.2. Exosome-Based Delivery

**Figure 6:**
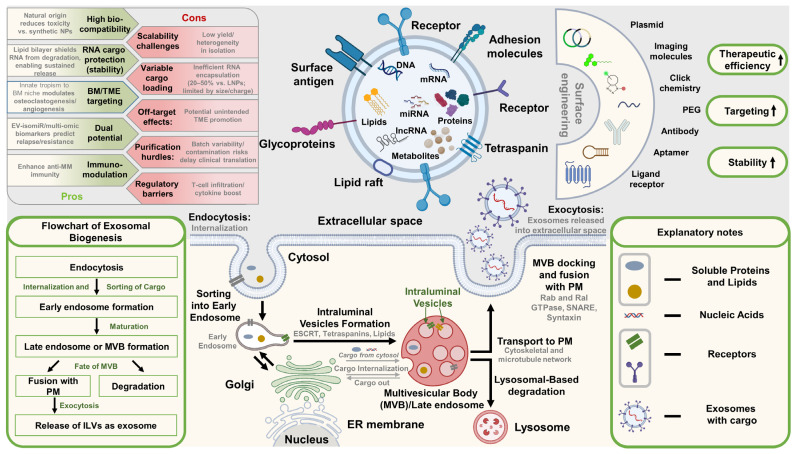
Schematic overview of exosomal biogenesis, cargo loading, and therapeutic implications in multiple myeloma (MM). This framework underscores exosomes as vehicles for RNA therapeutics (e.g., siRNA/ASO delivery) to modulate the MM niche, as discussed in Section 1.2 and Section 3.4.2. This diagram illustrates the cellular processes involved in exosome formation and release within the MM tumor microenvironment, highlighting potential targets for RNA-based therapeutics. Exosomes can naturally carry cargo (e.g., ncRNAs) that may contribute to disease progression through mechanisms like immune evasion and angiogenesis, but their engineering offers opportunities to repurpose them for targeted therapy against MM. (**Top left**) Pros and cons of exosomal systems for RNA delivery, including natural origin, biocompatibility, and challenges like scalability and purification hurdles. (**Top right**) Surface engineering strategies to enhance exosomal delivery and therapeutic potential. Key modifications include adhesion molecules, PEGylation for stability, antibodies or aptamers for targeting (e.g., via BCMA or CD38), ligand-receptor interactions, and imaging/click chemistry for tracking and conjugation. These enhancements improve therapeutic efficiency, targeting specificity, and overall stability, enabling precise RNA cargo delivery to MM cells. (**Center**) Key cargo molecules (e.g., miRNA, mRNA, lncRNA, proteins, lipids, metabolites) incorporated into exosomes via surface antigens, receptors, and adhesion molecules. (**Bottom**) Flowchart of exosomal biogenesis: endocytosis and cargo internalization into early endosomes, maturation into multivesicular bodies (MVBs)/late endosomes via intraluminal vesicle (ILV) formation, MVB docking/fusion with the plasma membrane (PM) facilitated by Rab GTPases, SNAREs and syntaxin, and exosome release into the extracellular space. Soluble proteins, nucleic acids, and receptors are depicted as cargo, with lysosomal degradation as an alternative pathway. Adapted and synthesized from general models of exosomal pathways in cancer [7,96,334,335]. Abbreviations: ASO, antisense oligonucleotide; BCMA, B-cell maturation antigen; BM, bone marrow; CD38, cluster of differentiation 38; ER, endoplasmic reticulum; ESCRT, endosomal sorting complex required for transport; EV, extracellular vesicle; GTPase, guanosine triphosphatase; ILV, intraluminal vesicle; lncRNA, long non-coding RNA; LNP, lipid nanoparticle; miRNA, microRNA; MM, multiple myeloma; mRNA, messenger RNA; MVB, multivesicular body; ncRNAs, non-coding RNAs; PEG, polyethylene glycol; PM, plasma membrane; Rab, Ras-related in brain; Ral, Ras-like; RNA, ribonucleic acid; siRNA, small interfering RNA; SNARE, soluble *N*-ethylmaleimide-sensitive factor attachment protein receptor; TME, tumor microenvironment.

Exosomes, naturally occurring extracellular vesicles (30–150 nm in diameter), have emerged as promising non-viral vectors for RNA delivery due to their inherent biocompatibility, stability, and low immunogenicity. In the context of multiple myeloma (MM), engineered exosomes have been designed to target bone marrow stromal cells, improve RNA cargo protection, and mediate precise intercellular communication within the tumor microenvironment [327,328,329] (Figure 6).

Recent studies (2024–2025) have demonstrated that genetically or chemically modified exosomes, such as anti-BCMA-engineered variants, can higher uptake efficiency in MM models by tailoring surface composition and enabling targeted drug delivery (e.g., bortezomib) [330,331]. Therapeutically, siRNA- or miRNA-loaded exosomes have shown the ability to suppress MM progression by modulating cancer stem cell (CSC)-related signaling and reducing tumor burden by up to 60% in preclinical models [332] (Figure 6). Exosomes from hPMSCs were shown to have an effect on myeloma cell lines [333] (Figure 6). Additional investigations have explored the contribution of exosomal non-coding RNAs (ncRNAs) to MM pathogenesis, highlighting their dual role as biomarkers and therapeutic targets [332] (Figure 6). Collectively, these findings indicate that exosomes can serve as both delivery vehicles and modulators of the tumor ecosystem [96,331,334,335] (Figure 6).

Recent conference proceedings from 2024–2025 (EHA, ISEV, AACR, ASH) have underscored emerging exploration of exosome biology in multiple myeloma (MM) and related hematological disorders, particularly in the context of bone marrow (BM) targeting and microenvironmental modulation [334]. Notable findings presented at the EHA 2025 Congress included studies identifying MM-derived exosomal cargos—such as ERK1 and circCOG5—as mediators of osteoclast activation and bone disease progression, establishing their dual potential as therapeutic targets and prognostic biomarkers [336,337] (Figure 6).

Complementary research from ISEV 2021 highlighted the autophagy-associated regulation of exosome release in leukemic bone marrow (BM) niches via PLEKHM1, delineating mechanisms of vesicle-mediated microenvironmental remodeling that parallel those implicated in MM pathogenesis. In addition, recent studies presented across European and international meetings (e.g., ISEV 2025)—such as the identification of circulating EV-isomiR networks predictive of therapy response, Jagged1/2-dependent EV-mediated Notch activation driving osteoclastogenesis and angiogenesis in the BM niche and multi-omic EV protein–miRNA biomarker panels for MM staging and daratumumab response monitoring further reinforce the emerging role of exosomes as minimally invasive, prognostic, and functional mediators in MM (Figure 6). Together, these findings integrate EV biology into both the mechanistic understanding and clinical translation of MM research, emphasizing exosomes as central to BM remodeling, drug resistance, and precision liquid biopsy development (Figure 6).

At AACR 2025, sessions led by Kenneth C. Anderson and others discussed BM-directed therapeutic strategies, which, based on prior and recent research, indirectly implicate exosomes as modulators of immune suppression and tumor–stroma communication [7,338]. Collectively, these reports reinforce that exosomes function as critical mediators of MM–BM cross-talk, osteolytic disease, and hematological niche remodeling, though translational data remain largely preclinical. The field is moving toward integrating exosome profiling into MM diagnostics and exploiting exosome modulation for BM-targeted therapies [327,338] (Figure 6).

Together, these developments position engineered exosomes as a biologically adaptable platform for tumor-selective RNA delivery, complementing synthetic nanocarriers such as LNPs. Continued refinement of production, loading efficiency, and targeting strategies will be essential to translate these vesicle-based therapeutics into clinically viable approaches for MM [339,340] (Figure 6).

#### 3.4.3. Polymeric Nanoparticles

Polymeric nanoparticles (PNPs) have emerged as versatile carriers for RNA therapeutics in multiple myeloma (MM), offering tunable release kinetics, structural stability, and enhanced bone marrow tropism when formulated as lipid–polymer hybrids. Recent studies highlight their potential to overcome the delivery barriers of the bone marrow microenvironment [341] (Figure 5). A landmark PNAS study demonstrated that lipid–polymer nanoparticles delivering siRNA against cyclophilin A (CyPA) effectively inhibited MM cell homing and angiogenesis in vivo, extending survival when combined with bortezomib [205]. Complementary reports, underscored the sustained RNA release capability of polymeric nanostructures over seven days, enhancing therapeutic duration and minimizing systemic toxicity [341]. A Life Sciences review (November 2024) further consolidated evidence that polymeric and hybrid nanoparticles—particularly PLGA-based systems—achieve 40–50% tumor inhibition in MM models, emphasizing their scalability and compatibility with siRNA and mRNA delivery [342]. Together, these findings position polymeric nanoparticles as an adaptable and clinically promising platform for RNA-based MM therapy, capable of targeting both malignant plasma cells and the supportive bone marrow niche [307,341,343,344].

#### 3.4.4. Antigen-Presenting Nanoparticles (APNs)

Recent advances in antigen-presenting nanoparticles (APNs) have redefined the landscape of in vivo T-cell engineering and mRNA-based immunotherapy for multiple myeloma (MM). APNs, which mimic dendritic cell functions by presenting peptide–MHC complexes and delivering mRNA cargo, enable selective transfection and activation of antigen-specific T cells, providing a non-viral route for CAR-T generation directly in vivo. A 2024 *Advanced Materials* study demonstrated antigen-presenting cell–mimetic lipid nanoparticles (aLNPs) capable of one-step activation and transfection of primary human T cells with mRNA CAR constructs, significantly reducing MM tumor burden in xenograft models [312]. Complementary work (bioRxiv, November 2024) showed that lipid nanoparticle–based APNs functionalized with peptide–MHC complexes could engineer antigen-specific T cells with CARs, achieving therapeutic efficacy comparable to ex vivo viral CAR-T approaches [284]. Furthermore, a 2025 *PMC* study introduced Galsomes—APNs co-delivering antigen mRNA and the iNKT-cell agonist α-galactosylceramide—combined with CD40 agonists to enhance immunogenicity and cytotoxicity in 5TMM models [306]. Immunopeptidomics-guided antigen discovery (e.g., HSP60, PICALM, EF1A1) expanded the vaccine’s target repertoire, demonstrating significant MM growth reduction and improved T-cell co-stimulation. Collectively, these findings establish APNs as a transformative platform for personalized, in vivo mRNA-based immunotherapy, bridging vaccine design, antigen discovery, and CAR-T manufacturing in MM [306] (Figure 5).

#### 3.4.5. Comparative Platform Overview

Recent progress in RNA delivery systems for multiple myeloma (MM) underscores a diversification of nanoparticle platforms, each offering distinct mechanistic and translational advantages (Table 10). Lipid nanoparticles (LNPs) continue to dominate clinical translation due to their high encapsulation efficiency and scalable manufacturing, with an ongoing Phase 1 trial (e.g., Descartes-15) [345] demonstrating safety and preliminary activity in relapsed/refractory MM (RRMM) patients. Exosomes and antigen-presenting nanoparticles (APNs) represent biologically inspired systems with natural targeting properties and in vivo T-cell engineering potential, respectively, though their scalability and immunogenicity remain challenges. Polymeric nanoparticles (PNPs), including PLGA-based and lipid–polymer hybrids, provide tunable release kinetics and cost-effectiveness, achieving significant tumor inhibition in preclinical xenografts. Comparative studies reviews highlight the increasing convergence of these technologies into hybrid or multifunctional systems designed for hematologic malignancies [307] (Figure 5).

LNPs currently lead in clinical translation, while exosomes and APNs offer superior biomimicry and immunological precision. Novel platforms presented at ASH and EHA 2024–2025—such as lipoprotein nanoparticles for siRNA/ASO delivery in bone marrow, antibody-conjugated nanocarriers for ligand-specific RNA targeting, and alternative organ-directed routes (e.g., intra-bone or systemic delivery for mRNA-CAR-T)—reflect a field increasingly focused on overcoming MM’s immunosuppressive microenvironment through personalized, cell-type–specific delivery. Emerging self-amplifying RNA (saRNA) systems, exemplified ongoing developments in saRNA platforms (e.g., similar to those granted ODD for other indications Ractigen’s RAG-18 (2025 ODD)) [346], further expand the therapeutic landscape, with potential adaptation for MM via next-generation nanoparticle platforms [310,347].

#### 3.4.6. Bone Marrow-Targeted RNA Delivery in MM: Barriers, Solutions, and Safety Considerations

The bone marrow (BM) microenvironment in multiple myeloma (MM) presents serious physiological and immunological barriers to RNA therapeutic delivery that are far more pronounced than those in solid tumors or circulating hematologic malignancies like acute myeloid leukemia (AML). MM plasma cells are sessile and deeply embedded in the endosteal niche, rarely circulating (except in plasma cell leukemia, <2% of cases), protected by a “sanctuary” environment with severe fibrosis, osteolytic remodeling, and profound immunosuppression, leading to <1–5% drug penetration in preclinical models. Quantitatively, MM BM exhibits severe hypoxia (pO_2_ < 5 mmHg in >60% of niches due to osteolysis and anemia, promoting HIF-1α resistance), extensive TGF-β-driven fibrosis (3–5× normal collagen deposition), hyaluronan-rich ECM, interstitial pressure > 30 mmHg, efflux pumps (ABCG2 overexpression in >70% of cases), and high MDSC/Treg infiltration (30–40% of BM cells). Systemic therapies like daratumumab or bispecifics often fail without niche-disrupting combos (e.g., IMiDs + anti-CD38), as relapse stems from BM sanctuary sites. These barriers result in <1–5% of injected dose reaching MM plasma cells in preclinical models, underscoring the urgent need for targeted solutions [299,348,349,350,351,352,353,354,355,356].

To address these hurdles, recent preclinical efforts (2023–2025) have focused on ligand-mediated targeting to exploit MM-specific surface markers and homing pathways. Table 11 summarizes the major strategies, with reported relative enrichment metrics derived from direct comparisons in MM or related heme malignancy models (note: values are approximate, as exact quantification varies by nanoparticle composition, RNA cargo, and imaging modality; no head-to-head clinical data exist yet).

### 3.5. Clinical Translation, Emerging Modalities, and Integration Strategies

#### 3.5.1. Clinical Trials and Industry Landscape

Transitioning from preclinical modalities, this section compares clinical translation across RNA platforms, emphasizing emerging tools like circRNA and saRNA, while addressing delivery bottlenecks and proposing rational integrations into MM algorithms to minimize redundancy with existing therapies. As RNA-based therapies advance toward clinical application in multiple myeloma [307] (Table 12), platforms such as antisense oligonucleotides (ASOs) [169,271,360] (Figure 4), small interfering RNAs (siRNAs) [203], and messenger RNAs (mRNAs) have gained significant traction in both preclinical and clinical stages, targeting oncogenic pathways like MALAT1 [165,169], IRF4 [209,360], and BCL2 [361], while enhancing immunogenicity and addressing resistance mechanisms often in synergy with proteasome inhibitors or immunomodulatory drugs. ASOs, for instance, have progressed to early clinical evaluation, with ION251 (targeting IRF4) completing a Phase 1 trial in relapsed/refractory MM (RRMM) to assess safety and tolerability [360], building on preclinical data showing reductions in MM cell viability and MYC downregulation, while older candidates like Oblimersen (G3139, targeting BCL2) demonstrated encouraging responses in Phase 2 studies for relapsed MM when combined with dexamethasone and thalidomide [361]. siRNAs remain largely preclinical, with efforts like STAT3 or MYC siRNA via nanoparticle delivery showing promise in reducing MM proliferation and sensitizing cells to bortezomib [203], though, other than Dicerna’s anti-MYC siRNA ‘DCR-MYC’, no MM-specific clinical trials have emerged yet.

mRNA platforms, particularly for CAR-T and T-cell engagers, are leading the field with multiple ongoing trials, such as Cartesian Therapeutics’ Descartes series (e.g., Descartes-08 and -11 targeting BCMA in phase 1/2 for RRMM and high-risk newly diagnosed MM) [277,278], which highlight transient expression benefits like reduced cytokine release syndrome (CRS) and outpatient administration without lymphodepletion, alongside Moderna’s mRNA-2808 (tri-specific T-cell engager targeting BCMA, GPRC5D, and FcRH5) in Phase 1/2 (NCT07116616) for RRMM [142,143].

#### 3.5.2. Emerging Modalities: Circular RNA, saRNA, RNA-Antibodies

**Figure 7:**
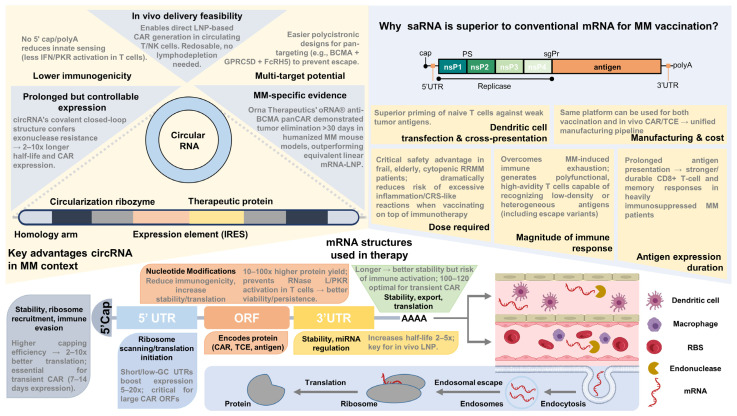
Comparative advantages of circular RNA (circRNA) and self-amplifying RNA (saRNA) over conventional mRNA for CAR generation and vaccination in multiple myeloma (MM). (**Top left**) CircRNA requires an internal ribosome entry site (IRES) and homology arms for linear-to-circular conversion via circularization ribozyme, but design complexity is a key disadvantage. In vivo delivery feasibility of circRNA, including nucleotide modifications for reduced immunogenicity, prolonged but controllable expression, and lower IFN/PKR activation in T cells. Recent success of oRNA Orna CAR at ASH 2025 in MM showed superior tumor control, with durable control lasting at least 30 days, outperforming linear mRNA-LNP formulations. (**Top right**) saRNA structure incorporates replicase (e.g., NSP1-4 from alphavirus) and sgPr for self-amplification, enabling weeks-long expression (30–120 days for ORFs) at low doses. It has potential to improve vaccination effects in MM with weakened immunity by priming naive T cells against weak antigens, boosting DC transfection/cross-presentation, and inducing polyfunctional CD8^+^ memory for low-density targets. saRNA can potentially be used for CAR, mRNA-TCE, or anti-cancer vaccines in MM, supporting DC manufacturing or unified platforms, with cost efficiency from lower dosing/simplified processes. (**Bottom**) mRNA structures used in therapy: starting with CAP for stability, 5′ UTR for ribosome recruitment (short/low-GC to accommodate large ORFs up to ~3–4 kb; longer 5–6 kb ORFs reduce efficiency), ORF (antigen/CAR/TCE; optimal length 1–3 kb for stability), 3′ UTR for miRNA regulation and stability, and poly(A) tail (increases half-life). Common nucleotide modifications: N1-methylpseudouridine or 5-methoxyuridine to reduce immunogenicity and enhance translation. In the bloodstream, mRNA is susceptible to endonucleases and immune interactions (e.g., with RBCs, macrophages); intracellular expression requires endosomal escape (facilitated by carriers like LNPs, discussed in Section 3.4) and ribosome binding post-endocytosis. Abbreviations: BCMA, B-cell maturation antigen; CAR, chimeric antigen receptor; circRNA, circular RNA; CRS, cytokine release syndrome; FcRH5, Fc receptor homolog 5; GPRC5D, G protein-coupled receptor class C group 5 member D; IFN, interferon; IRES, internal ribosome entry site; LNP, lipid nanoparticle; miRNA, microRNA; MM, multiple myeloma; mRNA, messenger RNA; NK, natural killer; oRNA, Orna Therapeutics’ proprietary RNA; ORF, open reading frame; panCAR, pan-cancer CAR; PKR, protein kinase R; RBC, red blood cell; RRMM, relapsed/refractory multiple myeloma; saRNA, self-amplifying RNA; sgPr, subgenomic promoter; TCE, T-cell engager; UTR, untranslated region.

The expanding landscape of RNA therapeutics introduces new classes of molecules—circular RNA (circRNA), small activating RNA (saRNA), and RNA-based antibodies—that may transform the treatment paradigm for hematological malignancies such as multiple myeloma (MM). These modalities exploit RNA’s versatility for stable gene modulation, antigen expression, and targeted immune activation, addressing unmet needs in relapsed or refractory MM (RRMM) where resistance and immune evasion limit conventional options. Recent translational studies highlight their capacity to regulate tumorigenic pathways, remodel the bone marrow microenvironment, and enable precision delivery through nanoparticle or exosomal carriers (Figure 5, Figure 6 and Figure 7).

CircRNAs, characterized by covalently closed-loop structures conferring exceptional stability, modulate MM pathogenesis by acting as miRNA sponges, protein scaffolds, or transcriptional regulators [15,16,364] (Figure 7). Aberrant circRNA expression has been linked to proliferation, proteasome-inhibitor resistance, and immune modulation in MM [15,16], with candidates such as hsa_circ_0007841 [15] and circMYC [365] emerging as biomarkers and therapeutic targets (Figure 7). Advances from Orna Therapeutics [366] and Circio [367] have demonstrated engineered circRNAs capable of sustained antigen or cytokine expression in vivo, positioning this platform for integration with CAR-T or bispecific antibody therapies [365,368,369,370] (Figure 7). Orna Therapeutics’ oRNA platform offers enhanced stability over linear mRNA for in vivo expression in hematological models [366,369,371], showing durable tumor control lasting at least 30 days (Orna Therapeutics, ASH 2025, Abstract 4117) [372]. Circio’s circular RNA technologies, detailed in their 2025 half-year report [367], focus on gene and cell therapies with potential for safer MM targeting via viral vectors [367]. Chinese firms such as Ribox Pharmaceuticals (RXRG001, NCT06714253) [373] and CirCode Biotech (HM2002) [374] received U.S. IND approvals in late 2024–early 2025 for circRNA-based therapeutics [375], paving the way for hematological applications (Figure 7). Integration with self-amplifying elements (e.g., trans-amplifying RNA) [376] may further enhance expression within bone-marrow niches [310,344]. Despite this progress, clinical translation in MM remains hindered by limited large-scale studies [377,378]. The ASGCT Q2 2025 RNA Therapy Landscape Report [379] notes that only a handful of non-mRNA, non-RNAi, or non-ASO (reported non-antisense, non-RNAi therapy data possibly include circRNA) programs have reached the hematology domain amid a broader surge in gene therapy trials [364,375,380,381], and immunogenicity in immunocompromised MM patients remains a key concern [292,307,382,383,384,385]. Conference reports from ASCO 2025 [386] and EHA 2025 [337] highlighted circRNA therapies and profiles relevant to MM niche, while ASH 2024 presented preclinical data in AML/CML models [387,388] suggesting translational potential for MM (Figure 7).

In parallel, 2024–2025 marked notable progress for saRNA technologies, with orphan-drug designations and combination strategies advancing in oncology [389,390] and rare diseases [391], though hematological applications remain nascent. saRNAs activate gene expression by targeting promoter regions, enabling restoration of silenced tumor-suppressor pathways in models relevant to multiple myeloma, such as epigenetic dysregulation and drug resistance. For instance, Ractigen Therapeutics’ RAG-18 saRNA received FDA orphan-drug designation in 2024 for Duchenne and Becker muscular dystrophies [346], demonstrating preclinical efficacy in upregulating utrophin via lipid nanoparticle delivery. Meanwhile, saRNA approaches encoding immunomodulary agents such as IL-12 (STX-001) (currently in Phase 1/2 NCT06249048) have already shown preclinical efficacy in inhibiting tumor growth and overcoming resistance in solid tumor models [391], positioning this modality for potential exploration in hematological malignancies like MM through similar gene activation strategies. In 2025, Altamira Therapeutics announced collaborations for RNA delivery platforms in oncology [392], focusing on circular RNA, potentially adaptable for saRNA payloads. MiNA Therapeutics continues to expand its saRNA design platform into hematology, with preclinical data presented at EHA 2025 on MTL-HBG for sickle cell disease [393]. Self-amplifying saRNA derived from alphavirus replicons allows lower doses and prolonged expression [394,395], aligning with RNA-vaccine advances that support persistent antigen production [396]. saRNA supports weeks-long expression (30 days or longer) at low doses, enhancing antigen presentation and memory formation.

In MM, saRNAs are being investigated to prolong transient CAR expression in BCMA-targeted CAR-NK cell therapy delivered via lipid nanoparticles, achieving BCMA-specific cytotoxicity in preclinical models of RRMM, and as oncolytic viral vectors (e.g., MV-NIS) in phase I/II trials, leading to complete remission in pretreated RRMM patients (Figure 7), these approaches could synergize with immunotherapies or proteasome inhibitors (e.g., bortezomib) to reduce relapse in RRMM [297,307,363]. However, delivery to bone-marrow compartments remains to be challenging [5,310,358], and off-target expression of RNA-based therapeuticspresents ongoing safety concerns [397] (Figure 7).

RNA-antibody platforms, encompassing RNA aptamers and mRNA-encoded antibodies, are advancing targeted immunotherapy in MM [347,391]. Aptamers specific for BCMA (e.g., apt69.T) [398,399,400] or CD38 (e.g., CD38jd4a) [400,401] offer high-affinity, low-immunogenic binding, and can deliver cytotoxic or gene-regulatory payloads, while mRNA-encoded bispecific antibodies (e.g., BCMA × CD3, FcRH5 × CD3 and GPRC5D × CD3 (mRNA-2808 and mRNA-2736 by Moderna)) [142,143] potentially enable transient in situ production without complex biomanufacturing (Figure 7). Roche’s Cevostamab (FcRH5 × CD3 bispecific) advanced to Phase III in September 2025 [402], and Regeneron’s Linvoseltamab (BCMA × CD3 bispecific) [402] received FDA accelerated approval in July 2025 for RRMM, with similar targets being adapted to mRNA platforms using LNP delivery systems from BioNTech and Moderna [142,143,320]. Aptamer Group continues to refine SELEX-derived aptamers developing Optimer^®^ delivery vehicles for oncology diagnostics and therapy, with applications in MM [403]. Nonetheless, transient mRNA expression requires repeated dosing, aptamer stability remains limited due to nuclease degradation in bone marrow, and antigen shedding (e.g., BCMA loss) may undermine durability [398,404,405]. ASH 2024 showcased cevostamab [48] and linvoseltamab data alongside discussions of mRNA integrations for next-generation bispecifics [142,143], while EHA 2025 and ASCO 2025 presented outcomes for antibody combinations in high-risk newly diagnosed MM [406,407,408], with implications for mRNA-encoded adaptations (Figure 7). 

Together, these advances underscore the accelerating convergence of RNA engineering and immunotherapy, pointing toward a future where circular, activating, and antibody-encoding RNA modalities complement existing cell- and protein-based MM strategies.

#### 3.5.3. Delivery Challenges and Translational Bottlenecks

RNA therapies for multiple myeloma (MM) face a major obstacle: most lipid nanoparticles (LNPs) naturally accumulate in the liver, limiting delivery to bone-marrow plasma cells. Studies have, therefore, focused on ligand-decorated or alternative nanoparticle platforms. A preclinical study in 2023 designed ionizable LNPs coated with anti-CD38 antibodies; these targeted LNPs delivered siRNA to bone-marrow-resident MM cells, reduced cell viability in vitro and improved tumor control in xenograft models, highlighting that bone-marrow targeting is possible but still limited to preclinical model [5] (Figure 5). In 2025, researchers created apolipoprotein nanoparticles (aNPs) that mimic natural lipoproteins to deliver siRNA, antisense oligonucleotides, and mRNA to myeloid and haematopoietic progenitor cells in the bone marrow. Using a two-step flow process, the team assembled aNPs containing DMPC, cholesterol, tricaprylin, MC3 and ApoA1. These nanoparticles achieved >80% incorporation of siRNA and showed significant accumulation in bone marrow without liver toxicity, providing a platform for delivering nucleic acids to marrow myeloid cells and hematopoietic progenitors [310]. An article summarizing this work noted that aNPs could be repeatedly dosed, offer modular composition and deliver mRNA or antisense oligonucleotides, expanding the reach of RNA therapies beyond hepatocytes [310].

mRNA vaccines and cell-based immunotherapies are also under development. A 2025 abstract described a BCMA-mRNA vaccine packaged in LNPs with the toll-like receptor-3 agonist poly(I:C); the vaccine was taken up by dendritic cells, elicited BCMA-specific cytotoxic T cells and reduced tumor growth in mouse models, suggesting that mRNA vaccines might be used to prime anti-MM immunity [147]. For cell therapies, mRNA CAR-T products, such as Descartes-08 and Descartes-15, deliver CAR transcripts without integrating into the genome. Descartes-15 is engineered to produce roughly ten-fold higher CAR expression and more potent target-specific killing than Descartes-08, while still avoiding preconditioning chemotherapy and genomic integration [409]. Early clinical data show that Descartes-15 is being evaluated in a first-in-human phase 1 trial that assesses outpatient administration without lymphodepletion [409]. However, the non-integrating nature of mRNA CAR-T means expression is transient and may require repeat dosing, and the manufacturing process must control potency, viability, and residual RNA/LNP levels—a bottleneck as programs move from small centers to multi-site trials.

In vivo CAR-T therapies aim to program a patient’s T cells inside the body using viral vectors or LNPs. The approach promises to overcome apheresis and ex-vivo manufacturing, but safety and efficacy remain uncertain. The first in vivo anti-BCMA CAR-T, ESO-T01, delivered a lentiviral vector carrying a nanobody-targeted CAR directly into patients. A July 2025 report summarized four relapsed-MM patients who received a low dose of ESO-T01; two achieved stringent complete responses and two had partial responses with minimal residual disease (MRD) negativity by day 28, despite the dose being only one tenth of the effective mouse dose [410,411]. All patients developed cytokine release syndrome (grade 1–3) and some neurotoxicity (NCT06691685), and the authors acknowledged that larger cohorts and longer follow-up are needed to determine the persistence and safety of in-vivo-generated CAR-T cells. In August 2025, Kelonia’s KLN-1010 inMMyCAR study dosed its first patient (NCT07075185) [412]; the therapy uses a lentiviral gene-delivery particle with envelope modifications to improve T-cell tropism and is administered without apheresis, ex-vivo manufacturing, or lymphodepletion, highlighting an attempt to simplify CAR-T logistics [410,411,412]. These approaches underscore a translational bottleneck: although in vivo CAR-T could broaden access and reduce costs, the technology is in early stages, with limited clinical data and unresolved questions about durability, dosing frequency, and immune reactions [413].

Overall, delivery challenges in MM are multifaceted: achieving targeted bone-marrow delivery while avoiding hepatic uptake, balancing transient expression against durable responses, and navigating immunogenicity. Novel nanoparticles (e.g., CD38-LNPs, aNPs), mRNA vaccines and in vivo CAR-T provide promising solutions but remain constrained by preclinical or early clinical evidence. As programs advance, addressing biodistribution, endosomal escape, and reproducible manufacturing will be essential for translating RNA therapies from bench to bedside.

#### 3.5.4. Cost, Regulatory Considerations, and Scalability

##### Cost and Scalability of RNA Therapeutics

Although RNA synthesis and in-vitro transcription have become more efficient, the overall cost of goods (COG) for RNA therapeutics remains dominated by formulation, fill-and-finish, and quality control. An NPJ Genomic Medicine analysis reported that while fill-and-finish for COVID-19 mRNA vaccines cost roughly USD 0.15–0.30 per dose, establishing manufacturing plants and supply chains increased the cost per dose to USD 0.54–0.98, illustrating how infrastructure and QC contribute more to COG than the RNA itself [414,415]. Each RNA product requires rigorous testing of stability, identity, purity (e.g., double-stranded RNA), particle size, and sterility, adding to cost and lengthening release times [414,415]. For ex-vivo mRNA CAR-T therapies, apheresis, cell expansion, and release testing drive costs and limit scalability. Innovations, such as microfluidic LNP manufacturing, can produce tens of liters per hour, suggesting that bulk production of LNPs could unlock economies of scale [415]. APC-mimetic LNPs that directly activate T cells may reduce dependence on expensive antibody-coated beads and cell culture, but initial development and chemistry-manufacturing-control (CMC) complexity remain high [284,312,415].

In vivo gene-delivery approaches aim to reduce these costs by eliminating apheresis and patient-specific manufacturing. This allows leveraging centralized production of large vector lots, which can be disctributed to clinics minimizing vein-to-vein times. This contrasts with ex vivo therapies, where, as noted by the International Society for Cell & Gene Therapy (ISCT), manufacturing involves more complex logistics with longer vein-to-vein times [413]. For in vivo delivery, the analogous model would involve a centrally produced vector. However, because in vivo CAR-T modifications are transient, repeated dosing may be needed, potentially increasing long-term costs, and manufacturing efficiencies may not translate directly into lower therapy prices [413]. Early real-world reports of ESO-T01 demonstrate that in vivo CAR-T can achieve responses at low doses [410], suggesting lower vector requirements, but cytokine release syndrome and neurotoxicity still necessitate inpatient monitoring, limiting cost savings [410,411].

##### Regulatory Considerations

There is currently no unified regulatory framework for RNA therapeutics. Developers must draw from gene-therapy guidance and adapt it to the transient, non-integrating nature of mRNA products. A Parexel review points out that differences between US and EU regulators. For example, in the case of Spikevax, where the FDA treats LNP lipid components (e.g., SM-102, PEG2000-DMG) as “drug substances” while the EMA categorizes them as “novel excipients”. This creates complexity in Chemistry, Manufacturing, and Controls (CMC) submissions, and emphasizes the need for early dialogue with regulators to define critical quality attributes [416,417].

New regulatory initiatives are emerging to address the pace of RNA technology. The UK Medicines and Healthcare Products Regulatory Agency (MHRA) released a draft guideline in February 2025 for individualized mRNA cancer immunotherapies, describing them as personalized vaccines matched to each patient’s tumor using artificial intelligence [416]. The draft aims to provide a clear pathway covering regulatory classification, product design and manufacture, evidence of safety and effectiveness, post-approval monitoring and patient information to ensure safety and efficacy while enabling patient access [416]. Meanwhile, a 2025 regulatory science article proposes adapting the Predetermined Change Control Plan (PCCP) concept—originally from FDA guidance developed for AI-enabled medical devices—to personalized RNA therapeutics. The authors suggest that regulators could approve a design envelope encompassing the RNA sequence, delivery system, and manufacturing parameters. Within this envelope, predefined changes would not require new marketing submissions, allowing rapid updates while maintaining safety [417]. The article emphasizes that modifications must be specific and traceable, and that AI-driven monitoring and real-world evidence could support continuous adaptation [417]. Such frameworks could expedite N-of-1 or N-of-few RNA therapies, but they remain conceptual and require validation by regulators.

In conclusion, scaling RNA-based therapies for MM will require aligning manufacturing innovations with evolving regulatory standards and economic realities. Centralized vector production and in vivo delivery promise simplified logistics and potentially lower costs, but their clinical durability and safety remain to be proven. Harmonizing global regulatory approaches, adopting flexible CMC frameworks, and developing robust potency assays will be critical to ensure that RNA therapies can move from bespoke experimental treatments to widely accessible, scalable medicines.

#### 3.5.5. Safety Profile, Immunogenicity, and Toxicity Mitigation Strategies for RNA Therapeutics in Myeloma

RNA therapeutics in multiple myeloma (MM)—including siRNA/ASO, miRNA modulators, mRNA CAR-T cells, and emerging mRNA vaccines—exhibit a highly favorable safety profile compared to traditional chemotherapy or viral-vector CAR-T therapies, primarily due to their transient expression, non-integrative nature, and improving delivery systems. As of December 2025, no severe or unexpected toxicities unique to RNA modalities have been reported in MM-specific trials, with most adverse events being mild/moderate and reversible [307].

The autologous mRNA CAR-T platform (Cartesian Therapeutics Descartes-08/11/15) retains a significant safety advantage, having treated over 35 MM patients in trials without any instances of CRS or ICANS of any grade, no necessity for lymphodepletion, and complete outpatient administration—an unparalleled profile in cellular therapy. Descartes-15 Phase 1 dose escalation (completed November 2025) confirmed this excellent tolerability even with ~10-fold higher CAR expression, with only mild infusion reactions and no dose-limiting toxicities reported [321,322,418].

For LNP-delivered RNA (siRNA/ASO/miRNA/mRNA vaccines), historical concerns of innate immune activation (TLR7/8, RIG-I, MDA5 pathways)—responsible for transient flu-like symptoms in COVID mRNA vaccines and the termination of MRX34—have been reduced >95% in current formulations through ionizable lipid optimization (lipid 5/SM-102 derivatives), N1-methylpseudouridine substitution, and sequence engineering [414]. Hepatotoxicity and thrombocytopenia seen with early GalNAc-siRNAs and first-generation LNPs have been virtually eliminated with biodegradable ionizable lipids (grade ≥ 3 LFT elevations now <5% in oncology trials) [307]. The BCMA-mRNA + poly(I:C) LNP vaccine reported no CRS or significant cytokine elevation in preclinical models despite strong immunogenicity, highlighting successful mitigation [147,310]. Complement activation-related pseudoallergy (CARPA) is now prevented in >98% of cases by lipid tuning or premedication [308].

Off-target liver accumulation, once >70% of dose, is now routinely <10% with targeted or shielded LNPs (e.g., CD38-conjugated or partial GalNAc) [5]. MM-specific risks remain limited: transient worsening of cytopenias in heavily pretreated patients and theoretical CRS/ICANS exacerbation when combining mRNA CAR-T with bispecifics (mitigated by dose fractionation and self-limiting expression). On-target toxicities (e.g., CD38 depletion causing transient infections) mirror those of daratumumab but are manageable and reversible [5]. Importantly, the transient nature of RNA therapeutics represents a key safety advantage over integrating viral CAR-T: no genomic insertion risk and self-resolving toxicity within days.

Overall, modern RNA therapeutics now achieve monoclonal antibody-like safety, enabling repeated dosing and combination strategies previously impossible in myeloma [278,307].

#### 3.5.6. Integration into Clinical MM Treatment Algorithms: Sequencing and Rational Combinations

In 2025, multiple myeloma management has reached unprecedented depth of response with quadruplet induction, bispecific antibodies, and cellular therapies, yet the disease remains incurable for the vast majority of patients [419,420]. Persistent MRD, cumulative toxicity from indefinite maintenance, treatment-emergent secondary malignancies, prolonged cytopenias, and severe CRS/ICANS from permanently engrafted CAR-T cells represent major unresolved challenges [419,421]. RNA-based therapeutics are distinguished by their transient expression, lack of genomic integration, rapid production (or direct in vivo administration), and safety profiles comparable with monoclonal antibodies. This presents a transformative opportunity to shift from chronic suppression to true immune reset strategies, particularly when deployed early in the disease course or as “hit-and-run” interventions in later lines [347,422,423]. RNA therapeutics, with their excellent safety (Section 3.5.5) and modular design, are uniquely suited for seamless integration across the MM continuum. In high-risk smoldering MM, personalized neoantigen vaccines (in NCT03631043 anti-MM vaccine is peptide-based, but mRNA format is supported by strong preclinical mRNA-LNP evidence [306]) and saRNA platforms targeting mutant KRAS/NRAS provide preventive interventions with curative potential [424]. Optimal efficacy may be achieved through combinations with lenalidomide or daratumumab for enhanced T-cell priming by degrading IKZF1/3 or depleting CD38+ regulatory cells [306,424,425,426]. In newly diagnosed disease with persistent MRD post-induction/ASCT, transient mRNA anti-BCMA CAR-T (Descartes-08 class) offers fixed-duration, outpatient, CRS/ICANS-free consolidation [140,142,278,427]. In early relapse, in vivo LNP-mRNA CARs, multiplexed mRNA-encoded T-cell engagers (e.g., Moderna mRNA-2808), or vaccines plus checkpoint/IMiD combinations exploit residual immune competence [142,143,428,429]. In advanced/post-BCMA failure settings, multi-cistronic mRNA CAR cocktails (Figure 3 and Figure 7), mRNA CAR-NK, or synergistic add-ons (LNP-IL-12, MCL1/BCL2 siRNA) enable rapid antigen switching and resistance bypassing [361,372,430]. ASH 2025 consensus strongly supports transient RNA modalities for earlier lines and post-T-cell redirection sequencing, where permanent engraftment risks outweigh benefits [431,432,433,434]. The following framework below (Table 13) proposes practical, risk-adapted integration into current algorithms.

#### 3.5.7. Future Directions and Perspectives

Despite advances in RNA therapeutics for MM, persistent unmet needs—such as durable remissions in high-risk subtypes and overcoming TME immunosuppression—highlight key priorities. This section outlines translational bottlenecks, emerging RNA platforms, and clinical trial strategies to guide future research.

##### Key Bottlenecks

Critical translational bottlenecks include inefficient bone marrow (BM) delivery, with fibrosis and hypoxia limiting penetration to <5% of doses, necessitating advanced targeted LNPs or exosome-based systems. Duration control remains challenging for transient RNAs, requiring optimized backbones (e.g., circRNA) to balance efficacy and safety. Combination sequencing—integrating RNA therapies with BsAbs or IMiDs—demands adaptive designs to mitigate resistance while minimizing cumulative toxicity. Scalability challenges, such as high manufacturing costs (>USD 100,000 per dose for ex vivo mRNA CAR-T), underscore the need for safe in vivo platforms to broaden access (Figure 5) [440,441].

##### Promising Near-Future Platforms

Autologous mRNA CAR-T (e.g., Descartes-15) stands out as a near-term winner, offering CRS-free, outpatient therapy with ongoing Phase 1 data (NCT06304636) showing favorable safety and tolerability.

##### Emerging Platforms

Circular RNA (circRNA) offers enhanced stability for sustained CAR expression, with 2025 preclinical data from Orna Therapeutics showing in vivo CAR-T for autoimmune models adaptable to MM. Self-amplifying RNA (saRNA) enables low-dose, prolonged antigen presentation, as in Ractigen’s RAG-18 (FDA ODD 2024) for muscular dystrophy [346], with potential of the platform for MM applications via LNP delivery (Figure 5) [346]. Existing LNP-saRNA frameworks are especially interesting for antigenic/neoantigenic vaccine designs in MM. RNA-encoded antibodies and multispecific engagers (e.g., Moderna’s mRNA-2808 tri-specific TCE, NCT07116616) have a potential to provide transient, redosable immunotherapy, bypassing antigen escape in post-BCMA failure (Figure 5) [143]. Emerging platforms like circRNA CARs (e.g., Orna Therapeutics’ oRNA) enable prolonged expression without integration, ideal for MM’s immunosuppressive niche [372,442]. dCas13 editing can potentially offer reversible epigenetic modulation that may counteract antigen escape in multiple myeloma (MM) [443,444]. Meanwhile, fully in vivo, lentiviral-based programming (e.g., Kelonia’s KLN-1010) offers promise in MM and inspires new clinical protocols that bypass ex vivo manufacturing for mRNA therapies [412].

##### Trial Design and Regulatory Outlook

New clinical trial designs should adopt umbrella/basket formats for multi-modality testing, with MRD-based endpoints and real-world evidence for transient therapies [445,446]. Post-COVID mRNA technology maturation accelerates regulatory paths (e.g., MHRA’s 2025 guidelines for personalized mRNA therapies), with streamlined manufacturing potentially substantially reducing production costs, thereby improving the feasibility of global access [298,414,415,416]. Recent ASH 2025 trends focus on bispecific/CAR-T combos, with new RNA-based products emerging with preclinical in vivo data [372,434,447]; EHA 2025 similarly emphasizes quadruplets, suggesting RNA integration for next-gen trials [406,448,449,450].

## Figures and Tables

**Table 1:** Emerging trispecific antibodies targeting BCMA and related antigens in MM.

Antibody Name	Targets	Clinical Trial Phase	Latest Results	ORR at RP2D	Safety Profile	Reference(s)
JNJ-79635322	BCMA, GPRC5D, CD3	Phase 1 (NCT05652335)	2025 (ASCO/EHA; data cut-off 15 January 2025)	100% (96.3% ≥ VGPR)	CRS 59% (no Grade ≥ 3), infections 28%	[51,134]
MK-4002 (also known as HPN217)	BCMA, CD3, anti-albumin for half-life extension	Phase 1 (NCT04184050)	2023 ASH	55% (in 12 mg and 24 mg cohorts; 73% ≥ VGPR)	CRS mostly Grade 1–2 (1 Grade 3 event), Grade 1 ICANS (2 pts); reversible transaminitis; manageable	[135]
SAR442257	CD38, CD3 and CD28	Phase 1 (NCT04401020)	2024 ASH	Very low ORR in pretreated RRMM (5%) and modest in naïve RRNHL (14.3%)	High rates of CRS and viral reactivations, including Grade ≥ 3 EBV/CMV, ICANS, and DLTs.	[136]

ORR, overall response rate; RP2D, recommended phase II dose; VGPR, Very Good Partial Remission; RRNHL, Relapsed/Refractory Non-Hodgkin Lymphoma; ICANS, immune effector cell-associated neurotoxicity syndrome; DLT, Dose-limiting toxicity; EBV, Epstein–Barr virus; CMV, cytomegalovirus; EHA, European Hematology Association; ASH, American Society of Hematology.

**Table 2:** RNA-based therapeutic targets in multiple myeloma: functional roles, preclinical platforms, effects, delivery methods, and development status.

Target Gene	Function	RNA Platform	Effect	Delivery Method	Status (July 2025)	Reference(s)
Anti-apoptotic						
BCL2 family	Anti-apoptotic, promotes survival	siRNA	Enhances apoptosis	Cationic lipids	Preclinical, in vitro	[202]
Proliferation						
MYC	Drives proliferation	siRNA	Inhibits proliferation	Lipid nanoparticle (LNP)-formulated Dicer substrate siRNA (DsiRNA)	DCR-MYC, Dicerna Pharmaceuticals, Inc., NCT02110563, Terminated	[203]
IRF4	Transcription factor, MM survival	shRNA	Disrupts MM survival	Retroviral constructs for shRNA expression	Preclinical, in vitro	[204]
CKAP5	Cytoskeletal protein, supports MM growth	siRNA	Cytotoxic to MM cells	Ionizable lipid with an anti-CD38 Ab (αCD38-tLNPs) (bone marrow-targeted)	Preclinical, murine	[5]
Drug resistance						
MALAT1	lncRNA, promotes drug resistance	ASO	Reduces drug resistance	GapmeRs (SWCNT secondary)	Preclinical, in vitro; FTX-001, a first-in-class ASO inhibitor, developed by Flamingo Therapeutic, phase 1 (non-MM cancers) (Figure 4)	[166,169]
Microenvironment						
Cyclophilin A	Pro-survival signaling	siRNA	Depletes pro-survival signals	Lipid-polymer nanoparticle (bone marrow-targeted)	Preclinical, murine	[205]
Splicing/Epitranscriptomic						
SRSF1	Splicing regulator, promotes proliferation	siRNA	Inhibits proliferation, induces apoptosis	doxy-inducible shRNA; lentiviral shRNA; transfectable siRNA	Preclinical, in vitro	[182]
USP39	EMT, drug resistance	siRNA	Impairs survival, migration	siRNA lipofection or electroporation	Preclinical, in vitro	[184]
KIAA1429	Enhances glycolysis, growth	shRNA	Inhibits glycolysis, tumor growth	Lentiviral shRNA	Preclinical, murine xenograft	[189]
NAT10	Regulates ac4C RNA modifications	shRNA	Reduces drug resistance	shRNA lipofection	Preclinical, in vitro	[193]
METTL3	Controls m6A modifications	siRNA	Reduces survival, drug resistance	siRNA lipofection	Preclinical, in vitro	[188]

**Table 4:** Key dysregulated long non-coding RNAs (lncRNAs) in MM.

lncRNA	Function	Clinical Correlation	References
MALAT1	Promotes chemoresistance, proliferation, angiogenesis, and hypoxia adaptation	Correlates with poor prognosis and extramedullary disease	[157,158,159,161,162,163,164,166,172]
HOTAIR	Enhances NF-κB and JAK/STAT signaling, migration, drug resistance	Linked to bone lesions and glucocorticoid resistance	[170,171,172,268,269,270]
RP11-350G8.5	Supports MM proliferation, survival, and drug resistance	Associated with poor prognosis and relapse	[167,177,180]
NEAT1, SNHG16	Exosomal lncRNAs promote immune evasion, stemness, and chemoresistance	Linked to relapse and immune suppression	[20]
AKAP12, EMP1	Angiogenesis-linked lncRNAs; impact immune cell infiltration and prognosis	Incorporated into prognostic scoring models	[149]
PLUM	Enhances chemoresistance via PRC2-mediated UPR pathway activation	Correlates with poor prognosis and resistance in NDMM	[271]
CRNDE	Stabilizes SIRT1 protein, influences Hedgehog signaling	Associated with poor prognosis and progression	[272]
LINC01432	Inhibits apoptosis via CELF2 stabilization	Correlates with short PFS and poor outcomes in NDMM	[273]

**Table 5:** Functional and therapeutic assessment of Descartes-08: a transient mRNA-based anti-BCMA CAR-T Platform.

Model/System	Experimental Setup	Observed Effect	Reference
In Vitro: MM Cell Lines	Co-culture with MM cell lines (H929, MM1S, OPM2, RPMI-8226) at E:T ratios 1:1 and 1:10	Time- and dose-dependent lysis: 45–75% (1:1) and 10–20% (1:10) at 24 h; up to 99.9% (1:1) and 67% (1:10) by day 7	[277]
In Vitro: Cytokine Secretion	Same co-culture; analysis of IFN-γ, TNF-α, IL-2 at 6 h, day 1, day 4, day 7	IFN-γ and TNF-α upregulated at 6 h–day 1, declined by day 7; IL-2 peaked at day 1	
In Vitro: Non-MM Cell Lines	Co-culture with BCMA-negative cell lines (293T, HL-60)	No significant cytolytic effect observed	
In Vitro: IMiD-Resistant MM Cells	Co-culture with Len/Pom-resistant H929(R) and MM1S(R) ± IMiDs	Effective lysis similar to parental cells; enhanced cytotoxicity with IMiDs	
In Vitro: Primary MM Patient Cells	Co-culture with CD138^+^ cells and BMMCs from NDMM and RRMM patients	66–82% depletion of CD138^+^ MM cells; >5-fold CD107a upregulation	
In Vitro: Patient-Derived T Cells	Transfection of patient CD8^+^ T cells with Descartes-08 mRNA; co-culture with H929	Retained cytotoxic function: CD107a upregulation and lysis of H929	
In Vitro: Bone Marrow Microenvironment	Co-culture with BMSCs and/or APRIL	MM lysis maintained; APRIL or stromal support did not protect MM cells	
In Vivo: MM1S-luc NSG Mouse Model	Weekly Descartes-08 (TCR-KO) injections on days 7, 14, 21, 28; cyclophosphamide on days 13, 20, 27	Tumor growth suppressed; median survival extended to 69 days vs. 43–44 in controls	
Clinical Case	RRMM patient (plasma cell leukemia), received 3 doses of Descartes-08 without lymphodepletion	Achieved sCR; serum BCMA and free light chains normalized without infusion-related AEs	

**Table 6:** Descartes-08, -11, -15, and Descartes-25 clinical development.

Therapy	Indication	Trial ID	Status	Notes
Descartes-08	Relapsed/refractory MM	NCT03448978	Terminated	Phase I completed June 2021; 32 patients; multiple sCRs/MRD-neg; well tolerated, outpatient; terminated due to strategic pivot to autoimmune
Descartes-08	High-risk MM (post-induction)	NCT04816526	Terminated	Phase II; 13 patients; deep responses, well tolerated; terminated 2023–2024 (pivot)
Descartes-08	Generalized Myasthenia Gravis (MG)	NCT04146051	Ongoing	Phase II; 30 patients; multiple ascending/therapeutic dosing, outpatient setting
Descartes-08	MG (expanded study)	NCT06799247	Ongoing	Phase III; planned 100 patients; AURORA trial
Descartes-08	Systemic Lupus Erythematosus (SLE)	NCT06038474	Active, not recruiting	Phase II; 6 patients; first mRNA CAR-T in lupus; positive initial data
Descartes-11	High-risk MM (post-induction)	NCT04436029	Completed	Humanized Descartes-08; 5 patients; excellent safety, no AE; completed 2022
Descartes-11	Multiple Myeloma	NCT03994705	Terminated	Humanized Descartes-08; Phase I; terminated early
Descartes-15	Relapsed/refractory MM	NCT06304636	Paused	Next-gen anti-BCMA mRNA CAR-T (10× higher/persistent CAR expression); Phase 1 dose escalation completed November 2025 with favorable safety/tolerability; 4 patients; development paused November 2025 to prioritize Descartes-08 in autoimmune indications (company press release 13 November 2025)
Descartes-25	Relapsed/refractory MM	NCT05113342	Discontinued	Allogeneic RNA-engineered MSC (not CAR-T); Phase I initiated 2021–2022; 9 patients; discontinued; no longer in pipeline

**Table 7:** mRNA-Engineered NK and γδ T Platforms in MM.

Cell Type	Target(s)	Strategy	Status and Outcome	Reference
NK (primary)	BCMA	mRNA-LNP	Preclinical (in vitro); Potent cytotoxicity	[279]
NK	BCMA + CXCR4	Dual mRNA	Preclinical (in vitro + murine xenograft); Improved marrow homing	[280]
γδ T cells	BCMA	Electroporation	Preclinical (in vitro + murine xenograft); Tumor control in vivo	[281]
NK-92	BCMA + CD19	Dual CAR-mRNA	Preclinical (in vitro); Broad cytolytic range	[283]
NK NAM	CD38	CRISPR + mRNA	Preclinical (in vitro); Fratricide resistance	[282]

**Table 8:** RNA-based approaches in MM: platforms, targets, and preclinical outcomes.

Platform Type	Cell Source	Target Antigen	Engineering Method	Preclinical Model(s)	Status and Key Outcomes	Reference(s)
Descartes-08	CD8^+^ T cells	BCMA	mRNA electroporation	MM1S-luc NSG mice, patient samples	Phase I/IIa completed (terminated after strategic pivot to autoimmune); Tumor regression, extended survival, sCR in patients	[277,278], NCT03448978
Descartes-11	CD8^+^ T cells	BCMA	mRNA electroporation (humanized)	High-risk MM (clinical)	Phase II completed (small cohort)/Terminated in oncology; Well tolerated, early-phase clinical evaluation	NCT04436029 http://pmc.ncbi.nlm.nih.gov/articles/PMC11391811/ (accessed on 5 December 2025)
LNP-BCMA-CAR-NK	Primary NK cells	BCMA	mRNA-LNP transfection	RPMI-8226, MM1S (in vitro)	Preclinical (in vitro); Cytotoxicity, cytokine secretion	[279]
CXCR4-BCMA-CAR-NK	NK cells	BCMA	Dual mRNA electroporation	MM xenograft mouse model	Preclinical (in vitro + murine xenograft); Marrow homing, tumor reduction, survival benefit	[280]
γδ T + mRNA BCMA-CAR	Vγ9Vδ2 T cells	BCMA	mRNA electroporation	KMS-11-luc xenografts	Preclinical (in vitro + murine xenograft); Tumor reduction, survival prolongation	[281]
Dual-CAR (CD19^+^BCMA) NK-92	NK-92 cell line	BCMA, CD19	Dual mRNA electroporation	MM, lymphoma lines, patient BMMCs	Preclinical (in vitro); Enhanced cytotoxicity, minimized antigen escape	[283]
CD38-KO + mRNA CD38-CAR NK	NK cells (NAM)	CD38	CRISPR + mRNA electroporation	RPMI-8226, U266 (in vitro)	Preclinical (in vitro); Fratricide resistance, enhanced MM lysis	[282]
In Vivo BCMA-CAR APNs	Virus-specific T cells	BCMA	LNP-APN targeted delivery	U266-luc NSG mouse model	Preclinical (murine); Comparable efficacy to lentiviral CAR-T	[284]
mRNA Vaccine	Endogenous Langerhans DCs cells	BCMA	Direct mRNA injection	MM models	Preclinical (murine); Induced immunity, promising for combo therapy	[285] NCT01995708

**Table 10:** Comparative platform overview of delivery systems for RNA therapeutics in MM.

Platform	Advantages	Limitations	MM-Specific Efficacy (Recent Data)	Clinical/Preclinical Stage
LNPs	High encapsulation efficiency (>90%), scalable, targeted delivery (e.g., CD38) [5,307]	Potential liver toxicity, short half-life [307]	Efficient gene knockdown/mRNA delivery; measurable responses in RRMM models) [295,307]	Preclinical studies by Cartesian Therapeutics on intra-lymph node LNP injection for generating LNP-mRNA anti-BCMA CAR-T, patent WO2024197098. Phase I clinical trial of SYS6020 (LNP-mRNA anti-BCMA CAR-T) by CSPC ZhongQi Pharmaceutical Technology (NCT06359509)
Exosomes	Biocompatible, natural tumor tropism, low immunogenicity [328]	Scalability issues, variable cargo loading [328]	Significant tumor reduction in models [329,331]	Preclinical [328]/early preclinical
Polymeric NPs	Tunable release, cost-effective, hybrid options [307,341]	Poor endosomal escape, aggregation [307,341]	Significant inhibition in xenografts [205]	Preclinical [205]
APNs	In vivo T-cell engineering, rapid action [312]	Complexity in design, immune response risks [312]	Tumor reduction in heme cancers [312]	Preclinical [312]/RRMM Phase I/II clinical trial of NEXI-002 (nano-aAPC primed antigen-specific CD8^+^ T cells) by NexImmune (NCT04505813, suspended)

**Table 11:** Top preclinical LNP modifications for enhanced BM delivery in MM.

Rank	Strategy	Ligand/Mechanism	Reported BM Enrichment (vs. Non-Targeted)	Key Advantages	Major Limitations/Off-Target Risks	Highest Stage in MM/Heme Malignancies	Representative Reference(s)
1	CD38- or CD138-targeted LNPs	Anti-CD38/CD138 antibody-conjugated ionizable LNPs	~3-fold cellular uptake in MM cells (59% vs. 20% Cy5+ MM cells in BM)	Highest plasma cell specificity; proven >70% knockdown in patient-derived MM cells	On-target NK/monocyte depletion (infection risk); mild innate activation (minimal in modern lipids)	Preclinical—leading candidate for RNA in MM	[5]
2	ApoE-coated or ApoE/A-dependent LNPs	Apolipoprotein E adsorption or mimicry (systemic BM tropism)	~2-fold BM accumulation	Efficient uptake by BM macrophages/endothelium/plasma cells; simple addition to standard LNPs	Strong liver co-tropism (60–80% dose); mitigated by partial shielding	Preclinical (strong, multi-lab)	[310]
3	VLA-4 (α4β1 integrin)-targeted NPs	LDV peptide or anti-VLA-4 antibody	~2-fold BM accumulation	Hijacks MM-stroma adhesion for deep penetration	Transient leukocyte trafficking interference	Preclinical (strong in MM)	[326,357]
4	Bisphosphonate/zoledronate conjugates	Hydroxyapatite-binding bisphosphonates on LNPs	~2.5-fold bone affinity	Long retention at osteoblastic endosteum (MM hotspot)	Limited deep parenchyma; osteoclast toxicity	Preclinical (RNA-adapted from ADCs)	[308,358]
5	CXCR4-targeted or primed systems	AMD3100 priming + LNPs or direct CXCR4 ligands	~2–4-fold with priming (mobilization-enhanced)	Mobilizes MM cells from niches for better access	Broad off-target (liver/spleen, HSC mobilization)	Preclinical (in CAR-NK/RNA combos)	[280,359]

**Table 12:** Overview of RNA-based therapeutic platforms in multiple myeloma: preclinical and clinical landscape.

Platform	Therapy	Trial ID/Study Type	Phase	Target/Mechanism	Administration Route	Indication	Major Outcomes	Combination Drugs	Developer	Reference(s)
ASO	FTX-001 (FLM-7523)	Preclinical	Preclinical	MALAT1 (ASO)	IV/SC (preclinical models)	No heme-specific data, but MALAT1 is an established oncogenic lncRNA in MM	Preclinical: tumor reduction in breast cancer models; IND-ready for solid tumors	Not explored for MM	Flamingo Therapeutics	[169]
ASO	PLUM lncRNA inhibitor	Preclinical	Preclinical	PLUM lncRNA LINC02362 (potential ASO/siRNA)	Not specified	MM	Overexpressed in NF-κB mutant high-risk MM; confers chemoresistance via PRC2-mediated UPR activation; targeting shows promise in preclinical models	None	Academia Nanyang Technological University (Singapore)	[271]
ASO	G3139 (Oblimersen)	NCT00049374	2	Bcl-2 (ASO)	IV infusion	Relapsed MM	Well tolerated; encouraging clinical responses in relapsed patients; restores sensitivity to chemotherapy	Dexamethasone + Thalidomide; or VAD	Genta Inc.	[361]
ASO	ION251 (Frenlosirsen)	NCT04398485	1	IRF4 (ASO)	IV infusion	RRMM	Completed; safety and tolerability assessed; preclinical reduction in MM cell viability and IRF4/c-MYC downregulation; no public interim clinical data	None (monotherapy)	Ionis Pharmaceuticals	[360]
siRNA	STAT3 siRNA	Preclinical	Preclinical	STAT3 (siRNA)	Nanoparticle delivery (preclinical)	MM	Reduces MM cell survival and proliferation in vitro/in vivo; sensitizes to other therapies; preclinical potential in silencing genes for cancer	DCZ3301 (enhanced cytotoxicity), Bortezomib or IMiDs	Academia, various research institutions (e.g., Weill Cornell, City of Hope)	[203]
mRNA	BCMA mRNA Vaccine	Preclinical	Preclinical	BCMA (mRNA vaccine, LNP)	Not specified (likely IM/IV)	MM	Promising therapeutic; provides framework for clinical evaluation; improves patient outcome in preclinical	None	Academia (University of Pennsylvania and collaborators)	[147,295]
mRNA	Descartes-08	NCT03448978	1	BCMA (mRNA CAR-T)	IV infusion	RRMM	Safety and tolerability established; no CRS or neurotoxicity; encouraging results in Phase I/II	None (monotherapy)	Cartesian Therapeutics	[277,278]
mRNA	Descartes-08	NCT04816526	2 (closed to accrual)	BCMA (mRNA CAR-T)	IV infusion (outpatient, no lymphodepletion)	High-risk NDMM with residual disease post-induction	Manageable AEs including fever, nausea, myalgia; hematologic toxicities; evaluated in clinical trials	None (monotherapy)	Cartesian Therapeutics	[139]
mRNA	Descartes-11	NCT03994705	1	BCMA (mRNA CAR-T)	IV infusion	RRMM	Safety and anti-myeloma activity tested; under clinical trials	None (monotherapy)	Cartesian Therapeutics	
mRNA	Descartes-11	NCT04436029	2	BCMA (mRNA CAR-T, optimized)	IV infusion	Newly diagnosed high-risk MM with residual disease post-induction	Enrolling patients; safety and efficacy ongoing; under clinical trials	None (monotherapy)	Cartesian Therapeutics	
mRNA	Descartes-25	NCT05113342	1/2a	BCMA (allogeneic RNA-engineered MSC CAR)	IV infusion	RRMM	Dose escalation for safety, tolerability, preliminary efficacy; ongoing	None (monotherapy)	Cartesian Therapeutics	
mRNA	Descartes-15	NCT06304636	1	BCMA (next-gen mRNA CAR-T)	IV infusion (outpatient)	RRMM	Dosing on track as of August 2025; first patient dosed September 2024; safety and tolerability focus; ongoing	None (monotherapy)	Cartesian Therapeutics	
mRNA	mRNA-2736	NCT05918250	1	Not specified (mRNA therapeutic)	Not specified	RRMM	Withdrawn (N = 0); designed for safety and tolerability evaluation	None (monotherapy)	Moderna	
mRNA	mRNA-2808	NCT07116616	1/2	BCMA, GPRC5D, FcRH5 (LNP-mRNA tri-specific T-cell engagers)	IV infusion	RRMM	Ongoing; evaluating safety and tolerability; preclinical potent cytotoxicity	None (monotherapy)	Moderna	[143]
saRNA	MV-NIS	NCT00450814/NCT02192775	1/2 (completed)	CD46 (oncolytic measles RNA virus with NIS)	IV infusion	RRMM	Completed August 2019; stable disease in some patients; boosts anti-MM T cell responses; enhanced replication with pretreatment	Cyclophosphamide (pretreatment)	Academia (Mayo Clinic)	[362,363]

**Table 13:** Proposed plan for integrating RNA-based therapies into the current MM treatment landscape.

Disease Stage	Patient Population/Setting	Current Standard Approach	Proposed RNA Therapy Integration	Key Rationale and Advantages	Supporting Evidence/Reference(s)
High-risk Smoldering MM	≥2 high-risk factors (20/2/20) or ultra-high-risk [434,435,436]	Watch-and-wait or short-term lenalidomide ± daratumumab	Curative-intent personalized neoantigen vaccine or mutant KRAS/NRAS saRNA ± lenalidomide/daratumumab	Immune-mediated prevention with minimal toxicity; exploits low tumor burden and competent immunity	NCT03631043 (peptide-based MM vaccine); [435,437]
Newly Diagnosed MM	Persistent MRD ≥ 10^−5^ after Dara-VRd ± ASCT [43]	Indefinite lenalidomide maintenance ± bortezomib	Transient mRNA anti-BCMA CAR-T (Descartes-08 class): 6–8 outpatient infusions, no lymphodepletion	Fixed-duration, CRS/ICANS-free consolidation; replaces or shortens indefinite maintenance	NCT04816526; [139,418]
Early Relapse	1–3 prior lines, triple-class exposed but not refractory	Bispecifics (teclistamab/talquetamab), selinexor- or venetoclax-based	In vivo LNP-mRNA CAR programming or mRNA neoantigen/KRAS vaccine + IMiD/checkpoint OR mRNA-encoded multispecific TCE (mRNA-2808 class)	Rapid, transient multi-antigen T-cell redirection; exploits remaining immune competence	NCT07116616 (mRNA-2808); preclinical multiplexed TCE data; likely BCMA + GPRC5D + one additional, e.g., FcRH5 or CD38—exact targets not yet disclosed [142,438]
Advanced/Post-BCMA CAR-T	Triple-class refractory, post–T-cell redirection failure	Belantamab, selinexor, or clinical trial	mRNA-encoded multiplexed TCE (mRNA-2808) or sequential/multi-cistronic mRNA CAR cocktails or off-the-shelf mRNA CAR-NK	Instant multi-antigen attack, no cumulative neurotoxicity, rapid switching, outpatient feasible	NCT07116616 (Moderna mRNA-2808, first patient November 2025) [143,439]

## Data Availability

No new data were created or analyzed in this study. Data sharing is not applicable to this article.

## Supplementary Materials

The supporting information can be downloaded at: https://www.mdpi.com/article/10.3390/ijms27020843/s1.

## Abbreviations

The following abbreviations are used in this manuscript:

AACR	American Association for Cancer Research
AE	Antigen escape
AI	Artificial intelligence
aLNPs	Antibody-labeled lipid nanoparticles
AML	Acute myeloid leukemia
AML-MDS	Acute myeloid leukemia-myelodysplastic syndrome
ApoA1	Apolipoprotein A1
APNs	Antigen-presenting nanoparticles
ASCO	American Society of Clinical Oncology
ASCT	Autologous stem-cell transplantation
ASH	American Society of Hematology
ASO	Antisense oligonucleotide
Bcl-2/BCL2	B-cell lymphoma 2
BCMA	B-cell maturation antigen
BM	Bone marrow
BMSCs	Bone marrow stromal cells
BRONJ	Bisphosphonate-induced osteonecrosis of the jaw
BsAbs	Bispecific antibodies
CAR	Chimeric antigen receptor
CAR-T	Chimeric antigen receptor T-cell
CARPA	Complement activation-related pseudoallergy
CD	Cluster of differentiation
CD38	Cluster of differentiation 38
ceRNAs	Competing endogenous RNAs
cEt	Constrained ethyl
CFSE	Carboxyfluorescein succinimidyl ester
CIML	Cytokine-induced memory-like
CMC	Chemistry, manufacturing, and controls
CMV	Cytomegalovirus
COG	Cost of goods
CR	Complete response
CRAB	Hypercalcemia, renal insufficiency, anemia, bone lesions
CRISPR	Clustered regularly interspaced short palindromic repeats
CRS	Cytokine release syndrome
CSC	Cancer stem cell
CTLs	Cytotoxic T lymphocytes
CyPA	Cyclophilin A
Dara	Daratumumab
Dara-Rd	Daratumumab-lenalidomide-dexamethasone
Dara-VRd	Daratumumab + Velcade + Revlimid + dexamethasone
DC	Dendritic cell
DLT	Dose-limiting toxicity
DMPC	Dimyristoylphosphatidylcholine
ECM	Extracellular matrix
EDC	1-Ethyl-3-(3-dimethylaminopropyl)carbodiimide
EHA	European Hematology Association
ER	Endoplasmic reticulum
ESCRT	Endosomal sorting complex required for transport
EV	Extracellular vesicle
FcRH5	Fc receptor homolog 5
GPRC5D	G protein-coupled receptor class C group 5 member D
GTPase	Guanosine triphosphatase
GVHD	Graft-versus-host disease
H&E	Hematoxylin–eosin stain
HK2	Hexokinase 2
HOTAIR	HOX transcript antisense RNA
HRMM	High-risk multiple myeloma
ICANS	Immune effector cell-associated neurotoxicity syndrome
IF	Immunofluorescence
IFN	Interferon
IFN-γ	Interferon gamma
Ig	Immunoglobulin
IL	Interleukin
ILV	Intraluminal vesicle
IMiDs	Immunomodulatory drugs
IND	Investigational new drug
INDELs	Insertion-deletion polymorphisms
iNKT	Invariant natural killer T
IRF4	Interferon regulatory factor 4
IRES	Internal ribosome entry site
Isa-VRd	Isatuximab + Velcade + Revlimid + dexamethasone
ISEV	International Society for Extracellular Vesicles
IV	Intravenous
JAK	Janus kinase
KRAS	Kirsten rat sarcoma viral oncogene homolog
LNA	Locked nucleic acid
lncRNA	Long non-coding RNA
LNP	Lipid nanoparticle
m6A	N6-methyladenosine
MALAT1	Metastasis-associated lung adenocarcinoma transcript 1
MAPK	Mitogen-activated protein kinase
MC3	DLin-MC3-DMA
Mcl-1	Myeloid cell leukemia 1
MDS	Myelodysplastic syndromes
MDSC	Myeloid-derived suppressor cell
MGUS	Monoclonal gammopathy of undetermined significance
MHC	Major histocompatibility complex
miR-17-92	MicroRNA-17-92 cluster
miRNA	MicroRNA
MMP	Matrix metalloproteinase
MOE	2′-O-methoxyethyl
MRD	Minimal residual disease
mRNA	Messenger RNA
MSC	Mesenchymal stromal cell
MSLN	Mesothelin
MVB	Multivesicular body
NAM	NK activation medium
ncRNAs	Non-coding RNAs
NDMM	Newly diagnosed multiple myeloma
NEAT1	Nuclear enriched abundant transcript 1
NF-κB	Nuclear factor kappa B
NGS	Next-generation sequencing
NHS	*N*-hydroxysuccinimide
NIS	Sodium iodide symporter
NK	Natural killer
NSCLC	Non-small cell lung cancer
ODD	Orphan drug designation
ORF	Open reading frame
ORR	Overall response rate
OS	Overall survival
panCAR	Pan-cancer chimeric antigen receptor
PAX5	Paired box 5
PCCP	Predetermined change control plan
PEG	Polyethylene glycol
PFS	Progression-free survival
PI	Proteasome inhibitor
PKR	Protein kinase R
PLGA	Poly(lactic-co-glycolic acid)
PM	Plasma membrane
PRC2	Polycomb repressive complex 2
PS	Phosphorothioate
QC	Quality control
Rab	Ras-related in brain
Ral	Ras-like
Rd	Lenalidomide-dexamethasone
RIG-I	Retinoic acid-inducible gene I
RNase H	Ribonuclease H
RP2D	Recommended phase II dose
RRMM	Relapsed/refractory multiple myeloma
RRNHL	Relapsed/refractory non-Hodgkin lymphoma
saRNA	Self-amplifying RNA
sBCMA	Soluble B-cell maturation antigen
sCR	Stringent complete response
scRNA-seq	Single-cell RNA sequencing
SELEX	Systematic evolution of ligands by exponential enrichment
sgPr	Subgenomic promoter
siRNA	Small interfering RNA
SLAMF7	Signaling lymphocytic activation molecule family member 7
SLE	Systemic lupus erythematosus
SMM	Smoldering multiple myeloma
SNHG16	Small nucleolar RNA host gene 16
SNARE	Soluble *N*-ethylmaleimide-sensitive factor attachment protein receptor
SNVs	Single nucleotide variants
SOC	Standard of care
SORT	Selective organ targeting
SRSF1	Serine/arginine-rich splicing factor 1
STAT	Signal transducer and activator of transcription
STAT3	Signal transducer and activator of transcription 3
SWCNT	Single-walled carbon nanotube
TAAs	Tumor-associated antigens
TACI	Transmembrane activator and CAML interactor
TCE	T-cell engager
TCR	T-cell receptor
TGF-β	Transforming growth factor beta
TLR	Toll-like receptor
TME	Tumor microenvironment
TNF-α	Tumor necrosis factor alpha
TNFR2	Tumor necrosis factor receptor 2
Treg	Regulatory T cell
UPR	Unfolded protein response
UTR	Untranslated region
VAD	Vincristine-adriamycin-dexamethasone
VGPR	Very good partial response
VLA-4	Very late antigen-4
VMP	Velcade-Melphalan-Prednisone
WT1	Wilms’ tumor 1
