# Supplementary material for: RNA-Based Therapeutic Strategies in Multiple Myeloma: From Molecular Targets to Delivery and Clinical Translation
Source: Int J Mol Sci. 2026 Jan 14;27(2):843. doi: 10.3390/ijms27020843 (PMC12841359; doi:10.3390/ijms27020843)
Supplement: Supplementary file 1 [file ijms-27-00843-s001.zip › Tables S1-S4_v4.pdf]

Supplementary Table S1. The function of lncRNA MALAT1 in Multiple Myeloma

MALAT1 Function	Effects in MM	Potential Biomarker Role	Therapeutic Targeting Strategy	Key References
Regulates MM cell proliferation and survival	Upregulation leads to increased cell growth and resistance to apoptosis	Correlates with poor prognosis and advanced disease stages	Antisense oligonucleotides (ASOs) and LNA gapmeRs	[1,2]
Enhances chemoresistance	Induces resistance to bortezomib and dexamethasone	Predicts response to bortezomib and dexamethasone	Combination therapy with proteasome inhibitors	[3,4]
Facilitates MM cell migration and invasion	Promotes MM progression and metastasis	Higher expression associated with worse overall survival	Targeting MALAT1 with siRNA/LNP formulations	[5,6]
Mediates hypoxia response and metabolic reprogramming	Supports MM survival under low-oxygen conditions	Hypoxia-driven MALAT1 upregulation linked to MM progression	Blocking MALAT1-mediated metabolic adaptation under hypoxia	[7,8]
Acts as a competing endogenous RNA (ceRNA)	Sponges tumor-suppressive miRNAs (e.g., miR-125b, miR-509-5p)	miR-125b/MALAT1 ratio may serve as a prognostic indicator	miRNA-mimic strategies targeting MALAT1 sponging functions	[9,10]
Regulates DNA repair and drug resistance	Interacts with DNA repair pathways (PARP1, LIG3) to maintain genomic stability	MALAT1 levels reflect DNA repair capacity and resistance to DNA-damaging agents	Combining MALAT1 inhibition with PARP1 inhibitors to disrupt DNA repair	[8,11]
Modulates tumor microenvironment and angiogenesis	Enhances interaction with stromal and endothelial cells to promote MM growth	MALAT1 expression in exosomes could serve as a liquid biopsy marker	Using MALAT1 inhibitors to reduce angiogenesis and MM-stromal interactions	[4,12]
Promotes extramedullary myeloma formation	MALAT1 expression increases following chemotherapy, linking it to stress-response mechanisms	Elevated MALAT1 levels indicate increased risk of extramedullary myeloma formation	RNA-based therapies to prevent chemotherapy-induced MALAT1 upregulation	[3,13]
Preclinical therapeutic development	Knockdown of MALAT1 leads to tumor regression in tumour models	MALAT1 suppression reduces MM cell viability in vivo	FTX-001: First-in-class MALAT1-targeting ASO in Phase 1 clinical trials for non-MM applications (Flamingo Therapeutics)	[14]

Supplementary Table S2. Experimental and clinical evidence for therapeutic targeting of lncRNA MALAT1 in Multiple Myeloma

	In vitro				In vivo				Clinical	
Reference	Cell Lines (In Vitro)	Silencing Method	Delivery Platform (In Vitro)	Effects Observed (In Vitro)	Model (In Vivo)	Therapy/Intervention (In Vivo)	Delivery Platform (In Vivo)	Effects Observed (In Vivo)	Patient Sample Info	Clinical Correlation
[1]	Not specified	—	—	No in vitro functional assays; focused on	—	—	—	No in vivo studies used	CD138+ cells from 5 normal, 9 MGUS, 33 SMM, 170	MALAT1 expression ↑ with disease stage; highest in PCL;

	In vitro				In vivo				Clinical	
				expression analysis					MM, 7 PCL patients	correlated with progression
[4]	RPMI8226, MM.1R, EA.hy926	siRNA-MALAT1 ± miR-15a/16 antagomir	Lipid-based (likely Lipofectamine)	↓ proliferation, ↑ apoptosis, ↓ angiogenesis; MALAT1 sponges miR-15a/16	RPMI8226 xenograft in nude mice	si-MALAT1 ± miR-15a/16 antagomir	Naked RNA agomir (intratumoral)	↓ tumor volume/weight, ↓ VEGFA, ↑ caspase-3	56 MM vs. 22 healthy (BM CD138+ cells)	MALAT1 ↑ in MM; correlated with ISS stage and poor OS; linked to VEGFA levels
[5]	OPM-2, U266	shRNA-MALAT1	Lentiviral vector	↓ proliferation, ↑ apoptosis; G0/G1 arrest; MALAT1 sponges miR-509-5p → ↑ FOXP1	U266 xenograft in nude mice	sh-MALAT1 vs. sh-NC	Pre-transduced cells (lentiviral)	↓ tumor volume, ↓ Ki-67, ↑ miR-509-5p	BM plasma cells from 37 MM vs. 19 healthy	MALAT1 and FOXP1 ↑; miR-509-5p ↓; MALAT1 positively correlated with FOXP1
[6]	NCI-H929, OPM-2	siRNA-MALAT1	Lipofectamine	↓ viability, invasion, glycolysis; ↑ apoptosis; MALAT1 sponges miR-1271-5p → ↑ SOX13	NCI-H929 xenograft in BALB/c mice	sh-MALAT1	—	↓ tumor volume/weight, ↓ SOX13, ↑ miR-1271-5p	Serum from 30 MM vs. 30 healthy	MALAT1 ↑, miR-1271-5p ↓, SOX13 ↑ in MM; MALAT1 linked to glycolysis/apoptosis
[7]	RPMI-8226, KMS-11, KMS-12-BM, MM.1S, U266	siRNA-MALAT1 and KDM3A under hypoxia	Lipid-based (likely Lipofectamine)	Under hypoxia, MALAT1 and KDM3A ↑; their knockdown → apoptosis and ↓ HIF-1α	—	—	—	No in vivo experiments used	BM samples from 15 MM patients	Hypoxia-induced MALAT1/KDM3A upregulation; KDM3A correlated with HIF-1α
[9]	NCI-H929, PRMI-8226	Lenti-miR-125b or sh-miR-125b; si-MALAT1; Notch1 overexpression	Lentiviral vector	miR-125b overexpression → ↓ MALAT1, Notch1, HES1; ↓ viability, ↑ apoptosis; Notch1 overexpression reversed MALAT1 knockdown effects	Subcutaneous xenograft (BALB/c nude)	Lenti-miR-125b	Pre-transduced cells	↓ tumor volume, ↓ Bcl-2, ↑ cleaved caspase-3	No patient samples used	Not applicable
[10]	JJN3, AMO-BZB	si-EZH2; EZH2 inhibitors (DZNep, GSK343, EPZ005687); MALAT1 ASOs	Lipid-based (likely Lipofectamine)	↑ miR-29b, ↓ H3K27me3 at miR-29a/b promoter; ↓ SP1, MCL-1, CDK6; MALAT1 knockdown showed same effect as EZH2 inhibition	—	—	—	No in vivo work	95 MM & 29 PCL datasets	EZH2 ↔ MALAT1 repress miR-29b
[11]	MM.1S, H929, RPMI8226	ASO-MALAT1 ± PARP1	—	↑ γH2A.X, ↑ cleaved PARP1, ↑ apoptosis; synergy with	Subcutaneous & disseminate	SWCNT-anti-MALAT1	SWCNT (nanotube-based)	↓ tumor growth, ↑ DNA damage	MM vs. healthy donor BM cells	High MALAT1 = poor prognosis

	In vitro				In vivo				Clinical	
		inhibitor (ABT-888) or bortezomib		bortezomib/ABT-888; MALAT1 binds PARP1/LIG3 promoting A-NHEJ repair	d MM in SCID mice			& apoptosis, ↓ Ki67, prolonged survival		
[12]	U266, MM1S, RPMI8226, RAW264.7	si-MALAT1, NSUN2, YBX1; MALAT1 overexpression; exosome transfer	Lipofectamine	Exosomes with MALAT1 promote osteoclasts via miR-17 sponging; NSUN2/YBX1-mediated m^5C modification stabilizes MALAT1	NOG mice injected with U266 cells	Exosomes ± MALAT1 knockdown	Exosome (natural vesicles) via tail vein	Bone lesions reduced by MALAT1 knockdown exosomes	36 MM vs. healthy BM samples	MALAT1/NSUN2/YBX1 ↑ in MM, correlated with bone damage & poor OS
[13]	H929, MM.1S	SWCNT-conjugated MALAT1 ASO	—	↓ MALAT1, ↑ apoptosis in MM cells	Disseminated MM in NOD SCID mice	IV injection of SWCNT-ASO	SWCNT (nanotube-based)	↓ tumor burden, prolonged survival, no toxicity	Not applicable	Not applicable
[2]	RPMI-8226, U266, MM.1S, NCI-H929	sh-MALAT1; miR-188-5p mimic/inhibitor	Lipofectamine	↓ viability, S-phase arrest, ↓ cyclinD1/E1, E2F1, p-Rb; ↑ apoptosis (↑ Bax, cleaved caspase-3/9; ↓ Bcl-2); co-transfection with miR-188-5p inhibitor rescues effects	U266 xenograft in nude mice	miR-188-5p agomir (intratumoral)	Naked RNA agomir	↓ tumor volume, ↑ apoptosis, ↓ proliferation markers (Ki67), ↓ cyclins, ↑ cleaved caspase-3	No patient samples analyzed	Cell lines selected based on endogenous MALAT1 and miR-188-5p levels
[3]	KMS12PE, KMS11, OPM2, RPMI8226, KMS11-BTZ, OPM2-BTZ	LNA GapmeR against MALAT1	Gymnotic (no transfection reagent)	MALAT1 upregulated by bortezomib, MG132, doxorubicin; silencing MALAT1 → no impact on proliferation or motility; correlated with HSP90A/B expression	—	—	—	No in vivo model used	114 MM patients (77 newly diagnosed, 37 relapsed), 48 MGUS, 19 lymphoma, 3 AML; 44 with extramedullary MM	High MALAT1 in EMM; correlated with del(17p) and poor prognosis
[8]	AMO-1, AMO-BZB, RPMI-8226, MM1S	LNA gapmeR antisense oligonucleotide (g#5)	Gymnosis (naked uptake)	↓ Viability, ↓ proliferation, ↑ apoptosis; ↓ proteasome subunit expression; ↓ NRF1/NRF2 via ↑ KEAP1; ↑ ROS, ↑ DNA damage	Murine xenograft model (AMO-BZB-luc cells)	g#5 treatment	Intraperitoneal injection (25 mg/kg)	↓ Tumor growth; ↑ caspase-3 activation; no systemic toxicity observed	Primary CD138+ MM cells from patients; PBMCs from healthy donors	MALAT1 upregulated in MGUS, SMM, and MM vs. normal PCs; no significant correlation with overall survival or time to relapse

Supplementary Table S3. The function of lncRNA HOTAIR in Multiple Myeloma

HOTAIR Function	Effects in MM	Potential Biomarker Role	Therapeutic Targeting Strategy	Key References
MM Cell Proliferation & Survival	Enhances MM proliferation via JAK2/STAT3 activation; knockdown induces cell cycle arrest at G0/G1 and apoptosis.	Elevated HOTAIR levels in serum and bone marrow correlate with advanced ISS stage and higher tumor burden.	siRNA/ASO knockdown to suppress JAK2/STAT3 signaling.	[15,16]
Drug Resistance	Drives dexamethasone (DEX) resistance via JAK2/STAT3; reduces apoptosis when overexpressed.	Serum HOTAIR levels predict resistance to glucocorticoids and proteasome inhibitors.	HOTAIR inhibition + JAK2/STAT3 inhibitors to restore DEX sensitivity.	[15]
NF-κB Activation	HOTAIR upregulates NF-κB expression, increasing MM survival and immune evasion.	Correlates with resistance to immunomodulatory drugs (IMiDs).	siRNA/ASO knockdown to suppress NF-κB activation.	[16]
Exosomal RNA Transfer & miRNA Regulation	HOTAIR functions as a miRNA sponge for tumor-suppressive miRNAs (miR-23b-3p, miR-27b-3p, miR-125b-5p), facilitating MM progression.	Exosomal HOTAIR levels could predict disease progression and microenvironmental remodeling in MM.	Exosome-modifying therapies to regulate HOTAIR-miRNA interactions	[17]
Bone Metabolism & Osteonecrosis (BONJ)	HOTAIR is upregulated in MM patients with bisphosphonate-induced osteonecrosis of the jaw (BONJ), suggesting a role in bone disease.	Potential risk biomarker for BONJ development in MM patients on bisphosphonate therapy.	RNA-based interventions to modulate lncRNA-related bone metabolism.	[18]
Prognostic Value	High HOTAIR expression potentially correlates with poor survival outcomes and increased relapse risk.	Potential marker for high-risk MM patient stratification.	HOTAIR-targeting RNA therapies combined with standard MM treatments.	[19]
Optimized Delivery Strategies	Ensuring effective delivery of siRNA/ASOs targeting HOTAIR in MM treatment.		Lipid nanoparticles (LNPs) and exosome-based delivery to enhance therapeutic efficacy.	[20]
Epigenetic Regulation via PRC2 Interaction	HOTAIR recruits PRC2 to silence tumor suppressor genes, promoting MM progression and chemoresistance	High HOTAIR expression correlates with poor prognosis and drug resistance	AC1Q3QWB: Small molecule inhibitor disrupting HOTAIR-PRC2 interaction, reactivating tumor suppressor genes (preclinical studies)	[21]

Supplementary Table S4. Experimental and observational insights into HOTAIR expression and silencing in Multiple Myeloma

Reference	Cell Lines (In Vitro)	Silencing Method	Delivery Platform (In Vitro)	Effects Observed (In Vitro)	Model (In Vivo)	Therapy/Intervention (In Vivo)	Delivery Platform (In Vivo)	Effects Observed (In Vivo)	Patient Sample Info	Clinical Correlation
[15]	MM.1S, U266,	shRNA-HOTAIR (via	Lipofectamine 2000 +	↓ Viability, G0/G1 arrest; ↓ DEX resistance via ↓ p-	—	—	—	—	118 MM patients, 78 healthy controls (serum,	HOTAIR significantly ↑ in MM serum, bone marrow, and CD138+

	MM.1R, JJN3	lentivirus)	lentiviral vector	JAK2/p-STAT3; ↑ apoptosis					bone marrow, CD138+ cells)	cells; AUC = 0.798 for MM diagnosis
[16]	Myeloma cell line (ATCC, not further specified)	siRNA-HOTAIR (HOTAIR knockdown)	Lipofectamine 2000	↓ HOTAIR → ↓ NF-κB protein, ↓ cell activity (MTT), ↑ apoptosis (Annexin V/PI), ↓ proliferation; HOTAIR promotes MM progression via NF-κB	—	—	—	—	None	Experimental model only; suggests HOTAIR is a potential oncogene in MM
[17]	U266 MM cells	siRNAs targeting HOTAIR, TOB1-AS1, MALAT1	Transient transfection	Knockdown of lncRNAs increased levels of miR-23b-3p, miR-27b-3p, and miR-125b-5p; overexpression of miR-214-3p and miR-5100 modulated MAPK, PI3K/AKT/mTOR, and p53 pathways, leading to increased proliferation and bortezomib resistance	—	—	—	—	None	Suggests lncRNAs in MM cells modulate exosomal miRNA transfer, influencing tumor progression and drug resistance
[18]	None used	None	Not applicable	Not applicable (observational only)	None	None	None	None	8 MM with BONJ, 8 MM without BONJ, 8 healthy donors (PB lymphomonocytes)	Downregulation of DANCR and MALAT1; upregulation of HOTAIR, MEG3, HOTTIP, etc. in BONJ; linked to impaired bone healing and angiogenesis
[19]	None used	None	Not applicable	Not applicable	None	None	None	None	Plasma samples from 62 MM patients (24 newly diagnosed, 23 post-therapy in CR/VGPR, 15 with PD/relapse) and 20 healthy controls	HOTAIR upregulated in newly diagnosed and PD/relapse MM patients compared to controls and CR/VGPR patients; correlated with bone marrow plasma cell percentage and ISS stage

References

1. Ronchetti D, Agnelli L, Taiana E, Galletti S, Manzoni M, et al. (2016) Distinct lncRNA transcriptional fingerprints characterize progressive stages of multiple myeloma. Oncotarget 7.
2. Liu H, Chi Z, Jin H, Yang W (2021) MicroRNA miR-188-5p as a mediator of long non-coding RNA MALAT1 regulates cell proliferation and apoptosis in multiple myeloma. Bioengineered 12: 1611-1626.
3. Handa H, Kuroda Y, Kimura K, Masuda Y, Hattori H, et al. (2017) Long non-coding RNA MALAT1 is an inducible stress response gene associated with extramedullary spread and poor prognosis of multiple myeloma. British Journal of Haematology 179: 449-460.

4. Yan H, Gao S, Xu A, Zuo L, Zhang J, et al. (2023) MALAT1 regulates network of microRNA-15a/16–VEGFA to promote tumorigenesis and angiogenesis in multiple myeloma. Carcinogenesis 44: 760-772.
5. Gu Y, Xiao X, Yang S (2017) LncRNA MALAT1 acts as an oncogene in multiple myeloma through sponging miR-509-5p to modulate FOXP1 expression. Oncotarget 8.
6. Liu N, Feng S, Li H, Chen X, Bai S, et al. (2020) Long non-coding RNA MALAT1 facilitates the tumorigenesis, invasion and glycolysis of multiple myeloma via miR-1271-5p/SOX13 axis. Journal of Cancer Research and Clinical Oncology 146: 367-379.
7. Ikeda S, Kitadate A, Abe F, Takahashi N, Tagawa H (2018) Hypoxia-inducible KDM3A addiction in multiple myeloma. Blood Advances 2: 323-334.
8. Amodio N, Stamato MA, Juli G, Morelli E, Fulciniti M, et al. (2018) Drugging the lncRNA MALAT1 via LNA gapmeR ASO inhibits gene expression of proteasome subunits and triggers anti-multiple myeloma activity. Leukemia 32: 1948-1957.
9. Gao D, Xiao Z, Li H-P, Han D-H, Zhang Y-P (2018) The mechanism study of miR-125b in occurrence and progression of multiple myeloma. Cancer Medicine 7: 134-145.
10. Stamato MA, Juli G, Romeo E, Ronchetti D, Arbitrio M, et al. (2017) Inhibition of EZH2 triggers the tumor suppressive miR-29b network in multiple myeloma. Oncotarget 8.
11. Hu Y, Lin J, Fang H, Fang J, Li C, et al. (2018) Targeting the MALAT1/PARP1/LIG3 complex induces DNA damage and apoptosis in multiple myeloma. Leukemia 32: 2250-2262.
12. Yu M, Cai Z, Zhang J, Zhang Y, Fu J, et al. (2024) Aberrant NSUN2-mediated m5C modification of exosomal LncRNA MALAT1 induced RANKL-mediated bone destruction in multiple myeloma. Communications Biology 7: 1249.
13. Lin J, Hu Y, Zhao J-J (2018) Repression of Multiple Myeloma Cell Growth In Vivo by Single-wall Carbon Nanotube (SWCNT)-delivered MALAT1 Antisense Oligos: 1940-087X. e58598 p.
14. Neveu M-AC, Ravenko A, Kim T-W, Luo X, Kim Y, et al. (2023) Abstract 450: Preclinical development of FTX-001: first in class inhibitor of the long non-coding RNA MALAT1. Cancer Research 83: 450-450.
15. Guan R, Wang W, Fu B, Pang Y, Lou Y, et al. (2019) Increased lncRNA HOTAIR expression promotes the chemoresistance of multiple myeloma to dexamethasone by regulating cell viability and apoptosis by mediating the JAK2/STAT3 signaling pathway. Mol Med Rep 20: 3917-3923.
16. Zhu BZ, Lin L (2019) Effects of lncRNA HOTAIR on proliferation and apoptosis of myeloma cells through NF-κB pathway. Eur Rev Med Pharmacol Sci 23: 10042-10048.
17. Saltarella I, Lamanuzzi A, Desantis V, Di Marzo L, Melaccio A, et al. (2022) Myeloma cells regulate miRNA transfer from fibroblast-derived exosomes by expression of lncRNAs. 256: 402-413.
18. Allegra A, Mania M, D'Ascola A, Oteri G, Siniscalchi EN, et al. (2020) Altered Long Noncoding RNA Expression Profile in Multiple Myeloma Patients with Bisphosphonate-Induced Osteonecrosis of the Jaw. BioMed Research International 2020: 9879876.
19. Shehata AMF, Kamal Eldin SM, Osman NF, Helwa MA (2020) Deregulated Expression of Long Non-coding RNA HOX Transcript Antisense RNA (HOTAIR) in Egyptian Patients with Multiple Myeloma. Indian J Hematol Blood Transfus 36: 271-276.
20. Chen Y, Li Z, Chen X, Zhang S (2021) Long non-coding RNAs: From disease code to drug role. Acta Pharm Sin B 11: 340-354.
21. Li Y, Ren Y, Wang Y, Tan Y, Wang Q, et al. (2019) A Compound AC1Q3QWB Selectively Disrupts HOTAIR-Mediated Recruitment of PRC2 and Enhances Cancer Therapy of DZNep. Theranostics 9: 4608-4623.
